# A stochastic view on surface inhomogeneity of nanoparticles

**DOI:** 10.1038/s41467-019-09595-y

**Published:** 2019-04-10

**Authors:** R. A. J. Post, D. van der Zwaag, G. Bet, S. P. W. Wijnands, L. Albertazzi, E. W. Meijer, R. W. van der Hofstad

**Affiliations:** 1 Institute of Complex Molecular Systems, Eindhoven University of Technology, P.O. Box 513, 5600 MB Eindhoven, The Netherlands; 2 Department of Mathematics and Computer Science, Eindhoven University of Technology, P.O. Box 513, 5600 MB Eindhoven, The Netherlands; 3 Department of Chemical Engineering and Chemistry, Eindhoven University of Technology, P.O. Box 513, 5600 MB Eindhoven, The Netherlands; 4 Department of Biomedical Engineering, Eindhoven University of Technology, P.O. Box 513, 5600 MB Eindhoven, The Netherlands; 5 Institute for Bioengineering of Catalonia, The Barcelona Institute of Science and Technology, 08028 Barcelona, Spain; 6 Present Address: DSM Coating Resins, P.O. Box 123, 5145 PE Waalwijk, The Netherlands; 7 Present Address: Department of Mathematics and Computer Science ‘Ulisse Dini’, University of Florence, 50134 Florence, Italy

## Abstract

The interactions between and with nanostructures can only be fully understood when the functional group distribution on their surfaces can be quantified accurately. Here we apply a combination of direct stochastic optical reconstruction microscopy (dSTORM) imaging and probabilistic modelling to analyse molecular distributions on spherical nanoparticles. The properties of individual fluorophores are assessed and incorporated into a model for the dSTORM imaging process. Using this tailored model, overcounting artefacts are greatly reduced and the locations of dye labels can be accurately estimated, revealing their spatial distribution. We show that standard chemical protocols for dye attachment lead to inhomogeneous functionalization in the case of ubiquitous polystyrene nanoparticles. Moreover, we demonstrate that stochastic fluctuations result in large variability of the local group density between particles. These results cast doubt on the uniform surface coverage commonly assumed in the creation of amorphous functional nanoparticles and expose a striking difference between the average population and individual nanoparticle coverage.

## Abstract (web summary)

Determining the spatial arrangement of molecules on a nanoparticle’s surface is key to understanding its interactions. Here, the authors use dSTORM imaging and probabilistic modelling to map the distribution of fluorophores on a nanoparticle, finding that ligand coverage is heterogeneous and highly variable between individual particles.

## Introduction

Control over multivalent interactions is essential in engineering various interactions between and with nanoparticles^1–5^. Multivalency—i.e. the simultaneous occurrence of multiple molecular recognition events between two entities—presents a ubiquitous principle for tuning a variety of interactions, due to the profound thermodynamic and kinetic consequences associated with interdependence of binding events^6–10^. The prevalence of multifunctional structures in living cells has prompted efforts to improve the understanding of multivalency in biological interactions^8,11^, and synthetic multifunctional scaffolds have attracted increasing attention as therapeutic agents^12,13^.

Multivalent interactions are highly sensitive to the local density and spatial arrangement of ligands^11,12,14,15^. As such, inadequate characterization of multifunctional structures is a major hurdle for the implementation of multivalency-based approaches in nanotechnology. The average functionalization density can easily be obtained using fluorescence assays or other ensemble methods, but these analyses provide no spatial information^16^. Additionally, the influence of stochastic variations in ligand concentrations, which has far-reaching consequences in biochemical signalling^17,18^, cannot be probed due to the averaging of signals inherent in these approaches. Hence, the average functionalization degree is not indicative for the surfaces of individual nanostructures in a population, and does not provide a reliable predictor for their properties.

Microscopic methods seem most suitable for providing a detailed representation of functional group topology on the single-scaffold level. For instance, the binding events in cellular interactions are separated on the nanoscale, and only few techniques can provide the spatial resolution required. Scanning probe microscopies (i.e. atomic force microscopy and scanning tunneling microscopy) provide very high resolution and have been applied in the study of nanoscale structures^19,20^. Progress has also been reported by using electron microscopy, in studies of epitope distributions in protein complexes^21^ and nanoparticles^22^. However, these approaches are generally invasive and lack chemical specificity, especially when multiple labels are required.

Super-resolution optical microscopy combines benign sample preparation with high sensitivity and specificity^23–26^, and as such provides perfect complementarity with the aforementioned microscopic techniques. Localization-based implementations provide high versatility and nanometre spatial resolution, and thereby present a unique capability to distinguish complex chemical features in a crowded molecular environment. Super-resolution images of biological and synthetic nanostructures have already facilitated several ground-breaking studies^27–32^, but provide intriguing challenges in terms of quantification^33–35^. For example, the finite localization precision combined with the reversible blinking of fluorescent dyes result in overcounting errors that have been approached using specific experimental methods (e.g. quantitative points accumulation in nanoscale topography (qPAINT)^36,37^) or using computational tools^38^. For photoactivatable dyes with short-lived dark states, time-correlation of individual localizations has been applied to diminish overcounting artefacts^39,40^. More generally, correlative approaches also reduce overcounting and provide an accurate indication of dye density, at the cost of local averaging of localizations^41,42^. As an alternative, probabilistic approaches can be used to address the challenge of fluorophore counting^43^, and several recent papers discuss the application of these methods in the context of direct stochastic optical reconstruction microscopy (dSTORM) and photo-activated localization microscopy imaging^44–47^.

The focus of existing literature is on quantifying dye (copy) numbers in well-separated clusters, thereby decoupling quantification and spatial analysis. Nevertheless, accurate estimation of both numerical and geometric factors is required to study realistic nanostructures with a range of interfluorophore distances, prompting a more thorough evaluation of spatial distributions based on the precision of individual localizations. A recently developed Bayesian clustering method has demonstrated the robust identification of localization clusters in a range of sample conditions, in both 2D and 3D^48,49^. However, this work focuses on separating molecule clusters from background noise, rather than on the structure of the molecular clusters, and the occurrence of multiple blinking in dSTORM hinders the application of these methods for quantitative dye location estimation.

In this work, we quantitatively analyse the functional group distribution on the surface of a multifunctional synthetic nanoparticle, using a combination of tailored photophysical modelling of the dye behaviour and Maximum Likelihood Estimation (MLE) of localization patterns in dSTORM. We assess the number of dyes in line with existing probabilistic approaches^43^, subsequently apply this information to estimate the dye locations based on the spatial modelling, and validate this approach using simulated datasets. The methodology is compatible with long-lived dark states common in dSTORM and PAINT microscopies, and fully exploits the high photon budget and repeated blinking inherent to these techniques. For prevalent amorphous polystyrene nanoparticles, we demonstrate large stochastic fluctuation in the quantities of functional groups attached to different particles in a population. Moreover, we establish spatial inhomogeneity of the attached moieties on individual nanostructures, representing an even larger variation in local dye density than can be explained by “random” attachment. Finally, we provide an empirical measure to quantify dye clustering on nanoparticles, allowing the customized prediction of interaction efficiency in a heterogeneous population.

## Results

### Addressing stochastic effects

**Fig. 1:**
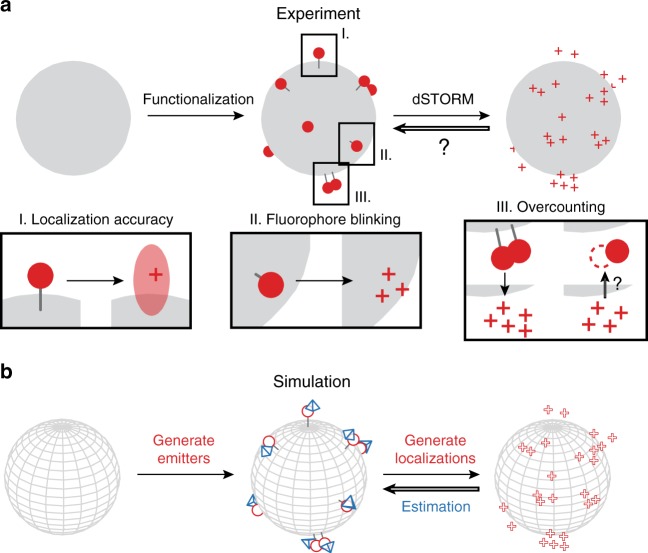
Creating a model for dSTORM-mediated analysis of functional group distribution. **a** Schematic depiction of the steps involved in the observation of fluorophore patterns on a spherical nanoparticle. Fluorescent dyes (red spheres) are, either chemically or physically, grafted to the nanoparticle (grey sphere) according to an unknown spatial distribution. The collection of localizations (red crosses) acquired during subsequent dSTORM imaging does not yield this spatial distribution exactly, due to a finite localization accuracy (I) and blinking of the dyes (II), leading to overcounting artefacts (III). **b** In our model, the steps mentioned in **a** can be mimicked in a fully controlled computational environment. The nanoparticle is functionalized with fluorescent emitters (red spheres) following an arbitrary spatial distribution. Subsequently, the dSTORM imaging process is simulated, leading to a collection of localizations (red crosses) with known sources. From these localizations, the positions of the emitters are estimated (blue pyramids). By comparing emitter locations and the corresponding estimates, the performance of the estimation procedure is assessed

The number of functional groups on a nanostructure cannot be observed directly from localization-based super-resolution images due to overcounting artefacts. Figure 1a schematically illustrates the dSTORM imaging procedure. First, reactive handles on the surface of a nanostructure are functionalized with photoswitchable dyes. During dSTORM acquisition, these dyes switch between short-lived fluorescent and long-lived dark states, and the emitted photons are captured by the camera. The centroids of the resulting diffraction-limited spots are fitted to obtain localizations, and a large collection of such localizations forms the dSTORM image. This fitting procedure has a finite resolution limited mostly by the brightness of the observed spot, resulting in an error in the calculated location of the centroid (I in Fig. 1a). Additionally, photoswitching of the fluorescent dyes occurs repeatedly and thus a single dye may yield multiple localizations over the course of an acquisition period (II in Fig. 1a). Hence, it is increasingly difficult to assign localizations to particular dyes in case multiple fluorophores are emitting in close proximity (III in Fig. 1a). The random nature of these photophysical processes represents an important obstacle in the determination of ligand densities.

In order to accurately analyse dye locations on densely functionalized nanoparticles, our analysis will rely on three properties: (i) the dye photophysics, including overcounting, need to be quantitatively described; (ii) the variability in the number of dyes attached to individual nanoparticles needs to be accurately captured; (iii) the spatial estimation algorithm must reliably reproduce the underlying (random, 3D and relatively dense) dye distribution from localization collections using (i) and (ii).

If properties (i), (ii), and (iii) are verified, and the dye dynamics are assumed not to change in the presence of other dyes, then the experimental distribution of dye labels on nanoparticles can be determined from dSTORM images. In this experiment, we apply an MLE procedure (vide infra) incorporating blinking behaviour and the uncertainties associated with individual localizations. Property (i) is validated by comparing the model fit with data from beads with a single-dye label attached (see section Photophysical modelling). Given (i), property (ii) is validated using data on the number of localizations on experimental beads (see section Nanoparticle functionalization). Lastly, property (iii) is validated using simulations, as illustrated in Fig. 1b (see section Spatial distribution of functional groups). In these simulations, fluorescent emitters are distributed over the surface of a sphere, the dSTORM imaging and localization process is mimicked, and the MLE procedure is applied to the resulting localizations. Since the underlying distribution of the emitters is known, the performance of the estimation procedure can be assessed, thus benchmarking our method.

### Photophysical modelling

**Fig. 2:**
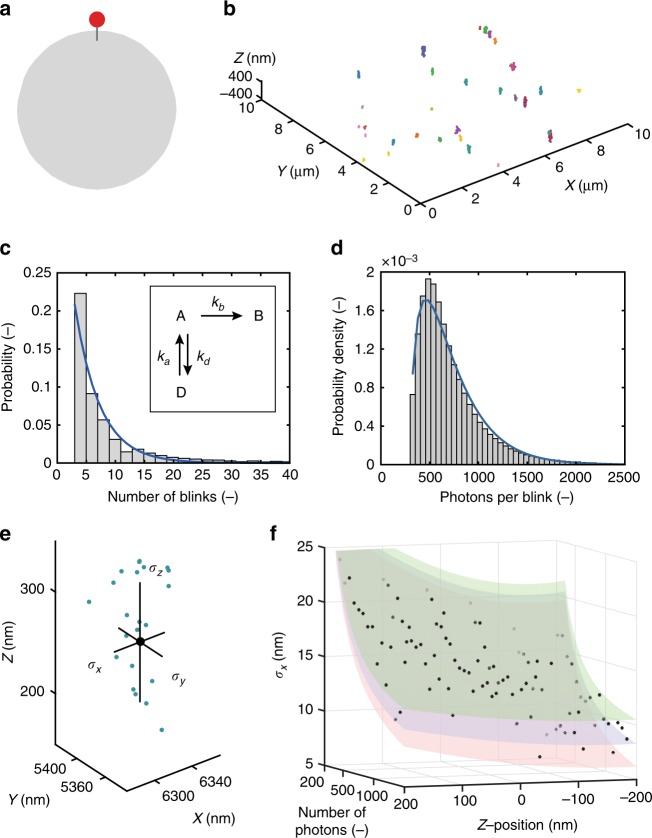
Analysis of dye photophysics through single-molecule experiments. **a** Schematic depiction of a mono-functional nanoparticle, obtained by reacting a large excess of carboxyl-functional polystyrene beads (diameter 330 nm) with a dilute solution of Alexa647 fluorophore. Experimental details can be found in the Methods section. **b** dSTORM image of mono-functional nanoparticles. Nanoparticles were immobilized on a glass coverslip and imaged using astigmatic 3D-dSTORM. **c** Probability distribution of the number of blinks per dye (grey bars), $n_{{\mathrm{dyes}}} = 2206$. The probability distribution has been fit using a conditional geometric distribution (blue line), ${\mathrm{Geometric}}\left( {\hat p_{{\mathrm{bleach}}} = 0.21} \right),$ corresponding to a three-state model. Inset: three-state model for reversible photoswitching in dSTORM imaging, including active (A), dark (D), and bleached (B) states and the corresponding transitions. **d** Probability density distribution of the number of photons per blink, i.e. period in the active state, (grey bars), $n_{{\mathrm{blinks}}} = 2.6 \times 10^6$. The probability density distribution has been fit using a conditional gamma distribution (blue line), ${\mathrm{Gamma}}\left( {\hat k = 1.62,\hat \theta = 3.84 \times 10^{ - 3}} \right)$. Details of the distributions used in **c** and **d** can be found in the Supplementary Methods. **e** Determination of experimental localization accuracy. Localizations corresponding to a single dye are indicated as green points, and the localization errors *σ*_*x*_, *σ*_*y*_, *σ*_*z*_ of a particular centroid are the distances (in the *x*, *y*, *z* dimensions, respectively) between this centroid and the average of all centroids corresponding to the same dye (black point). **f** Dependence of the experimental localization error *σ*_*x*_ on the number of photons of the localization and the estimated z-position of the dye. Black points indicate the average estimated absolute error (i.e. a measure for the experimental error of the localization process) for all localizations combined in 75 nm ×75 photon bins. Transparent surfaces indicate the localization errors predicted by different photophysical models: a basic model of the 3D fitting error (red), an extended model including fiducial marker-based drift correction (blue), and a complete normal-normal mixed model further accounting for point spread function (PSF) deformation (green). Similar graphs for the localization error in the *y* and *z* dimensions, and the width error in the *x* and *y* dimensions, are shown in Supplementary Figs. 7–9

The single-molecule dynamics of the Alexa647 fluorophore on a nanoparticle surface have been characterized to quantify the relevant photophysical processes. Carboxyl-functional polystyrene nanoparticles (diameter 330 nm) functionalized with a single Alexa647 moiety (Fig. 2a, experimental details are described in the Methods section) have been immobilized to a glass coverslip, and imaged using astigmatism-based 3D-dSTORM (Fig. 2b). The resulting localization clusters correspond to a single fluorophore photoswitching over the course of a dSTORM acquisition, providing a suitable dataset to verify property (i).

Inspired by existing models for photoactivatable dyes^39^, we apply a three-state model for photoswitchable dyes (Fig. 2c). Such a competition between processes with constant transition probabilities results in a geometric distribution for the number of active periods (i.e. a complete period between thiol-mediated or irreversible dark states) before bleaching, experimentally equating the number of localizations per dye. Indeed, the observed localizations can be adequately fit using a geometric distribution, as shown in Fig. 2c.

Since the number of photons in a localization determines the accuracy of its centroid fit^50,51^, this is a crucial parameter to quantify the spatial aspects of fluorophore distribution. Previous single-molecule studies have shown that fluorophores do not emit constantly over an active period, but alternate short-lived emissive and non-emissive states within this period^52–54^. Assuming exponential residence times in the emissive state, the total number of photons emitted is well approximated by a gamma distribution, which can indeed adequately fit the data (Fig. 2d).

Individual localizations are modelled as a 3D Gaussian distribution around the real location of the dye, with variances depending on the number of photons. Hence, the 3D spread of localizations that originate from a single fluorophore is used as an experimental measure of localization uncertainty (Fig. 2e). Figure 2f shows an overlay of the experimental localization uncertainty (solid points) with different variance expressions. We extended the theoretical expressions derived by Mortenson et al.^55^ for the estimation errors of a 2D Gaussian mask estimator for the centroid location to astigmatic 3D-imaging (red surface). The considerable deviation to the experimental data indicates that this basic framework fails to capture all relevant processes. If the expression is extended to include the effects of drift correction, a better approximation is obtained (blue surface). Further enhanced prediction of experimental data is provided by incorporating variability related to optical effects—for example, coupling of the fluorophore excitation dipole to nearby interfaces^56^. The resulting localization-dependent distortion of the PSF may be captured by replacing the Gaussian distribution by a normal-normal mixture model, and the complete model provides a further improved estimate of localization errors in three dimensions (green surface).

### Nanoparticle functionalization

**Fig. 3:**
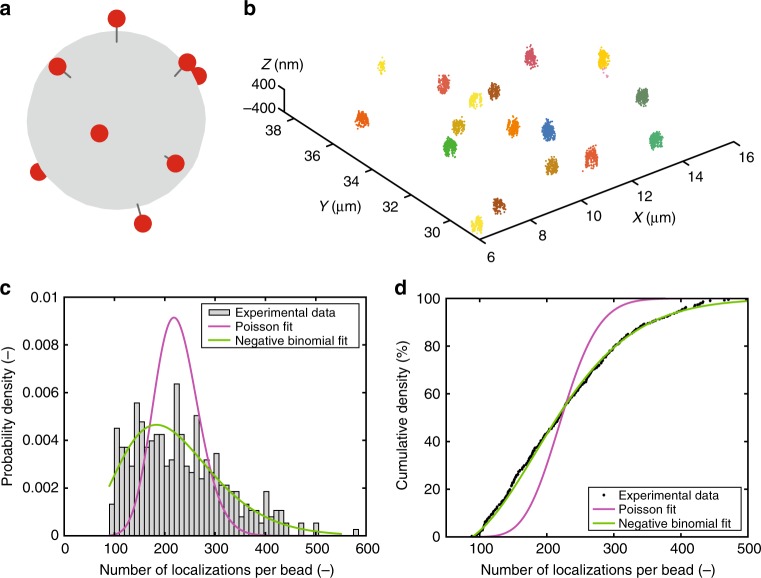
dSTORM imaging of dye-functionalized nanoparticles. **a** Schematic depiction of a spherical nanoparticle, functionalized with multiple Alexa647 fluorophores. The average density of the fluorophores can be adjusted by varying reaction conditions. **b** dSTORM image of multifunctional nanoparticles. Medium-density nanoparticles were immobilized on a glass coverslip and imaged using astigmatic 3D-dSTORM. **c** Probability mass function of the number of localizations per nanoparticle (grey bars), $N_{{\mathrm{beads}}} = 379$. The probability mass function has been fit using a conditional compound Poisson distribution (solid magenta line), ${\mathrm{Poisson}}\left( {\hat \lambda = 46.82} \right),$ representing the functionalization reaction as a completely random arrival process. An alternative fit has been performed using a conditional compound negative binomial distribution (solid green line), ${\mathrm{NBinomial}}(\hat r = 6.54,\hat p = 0.12)$, indicating a functionalization process with increased dispersion. **d** Cumulative probability distribution of the number of localizations per bead (solid points). The plot shows an improved quality of the fit for a compound negative binomial function (solid green line) compared to a compound Poisson function (solid magenta line). Details of the distributions used in **c** and **d** can be found in the Supplementary Methods

For the purpose of analysing the distribution of functional groups on the surface of a nanoparticle, and in particular the influence of stochastic variability, multifunctional dye-appended 330 nm polystyrene beads have been created (Fig. 3a). Functionalization has been performed using amine-NHS chemistry in dilute solution (nanoparticle and experimental details can be found in the Methods section), a common procedure for creating therapeutically active nanoparticles. By adjusting the reaction conditions, nanoparticles with different average degrees of functionalization (low, medium, high) could be generated in a simple manner. Medium-density particles have been imaged using astigmatic 3D-dSTORM (vide supra), resulting in well-reconstructed spherical clusters of localizations (Fig. 3b).

A measure of the variability between nanoparticles in a population can be obtained by investigating the distribution of the number of localizations per bead. In Fig. 3c, the probability mass function for this number of localizations is depicted (grey bars). This distribution is determined by the interplay between two properties: the number of fluorophore molecules attached to a nanoparticle, and the number of localizations per fluorophore. The latter is geometrically distributed with known parametrization (Fig. 2c). For fully random attachment processes, in which fluorophore molecules in the reacting solution have an equal probability to attach to any nanoparticle, the former property is expected to follow a Poisson distribution. Combining these distributions results in a compound Poisson distribution, which has been fit to the probability mass function in Fig. 3c (red solid line). As can be clearly seen, a compound Poisson distribution does not provide an appropriate fit. However, when the functionalization process is not Poissonian, but is characterized by additional dispersion instead, the number of dyes per nanoparticle can be captured by a negative binomial distribution. Then, the observed number of localizations per nanoparticle equals a random sum of negative binomial random variables, referred to as a compound negative binomial. This tailored distribution successfully captures the width and asymmetry of the data (Fig. 3c, solid green line) and thus verifies property (ii). The improved quality of the fit can be clearly seen in the cumulative distribution functions (Fig. 3d), demonstrating larger variability than expected by traditional count statistics. This overdispersion may indicate dispersity on the bead level or dependence between the reactions of different fluorophore molecules on a particular bead, thus casting doubt on the assumption of fully random functionalization that is almost universally adopted in the field.

### Estimating fluorophore locations

**Fig. 4:**
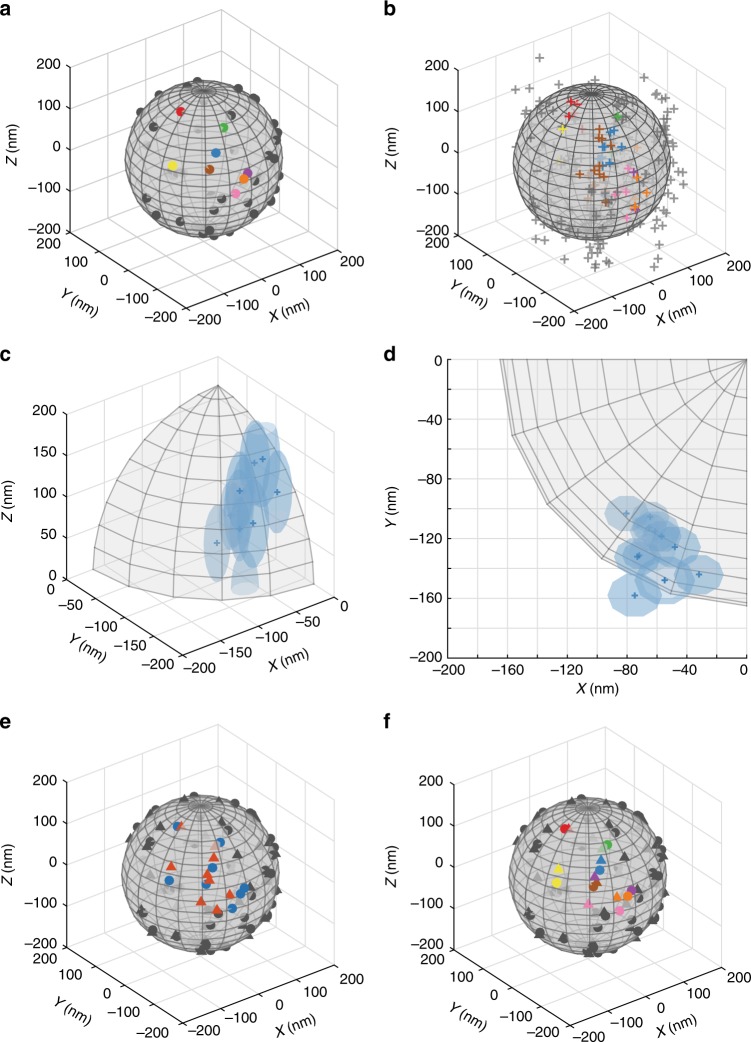
Model-based validation of MLE for the fluorophore distribution on a single nanoparticle. **a** Simulated fluorophore locations (solid points) on a spherical nanoparticle (grey sphere) with a stochastically homogeneous spatial distribution. The number of fluorophores is a realization of the introduced negative binomial distribution. The spatial locations are realizations of a uniform distribution over the surface of the sphere. **b** Simulated clusters of localizations (crosses) based on the generated fluorophore locations in Fig. 4a. The simulations of the dSTORM imaging process are generated realizations of the probabilistic model described in detail in the Supplementary Methods. **c** Measurement uncertainties for each localization, dependent on brightness and z-position. The uncertainty is graphically depicted as an ellipsoid of one standard deviation in three dimensions (transparent surface). Only localizations associated with the fluorophore colour-coded in blue are depicted. **d** Measurement uncertainties viewed along the *z*-axis. **e** Final MLE estimates of the fluorophore locations (solid triangles), resulting from the EM-algorithm based on the simulated localizations and associated uncertainties. The actual locations of simulated fluorophores are indicated for reference (solid points). **f** Matching of estimated and actual simulated fluorophore locations, using the Hungarian algorithm given the real number of dyes. Matched pairs are indicated by identical colours. The average distance between pairs is a measure of the estimation error in the MLE procedure

Not only the number of functional groups can vary between different nanoparticles, but also their spatial distribution on the particle surface. A statistical procedure has been developed to analyse the distribution of fluorophores on a nanoparticle, based on clusters of localizations as present in a dSTORM image. Since the actual locations of fluorophores are unknown in an experimental setting, the performance of this procedure has been verified using stochastic simulations (Fig. 4), validating property (iii). In these simulations, fluorophores are placed on the surface of a nanoparticle according to a pre-defined distribution (Fig. 4a). Subsequently, the dSTORM imaging process is mimicked, resulting in a localization-based image (Fig. 4b). Using this simulated image as input, first the most likely number of dyes based on the compound negative binomial distribution is calculated using the number of localizations (vide supra).

We then approximate the locations of the source fluorophores using MLE, a singular and essential step for the determination of functional group distributions. In brief, the k-means algorithm^57^ is applied to provide an initial guess of the source locations. Based on this initial estimate, the probability distribution of origination (i.e. the chance that a localization originates from a particular source) is derived for all localizations using MLE. Subsequently, an improved location estimate is computed using weighted contributions of localizations, taking into account the distribution of origination, the nanoparticle geometry and the measurement uncertainty (Fig. 4c, d). This last factor enables differential appraisal of bright blinks with a high localization precision, and localizations based on few photons with a larger error margin. Allocation of localizations and weighted displacement of sources are then iteratively repeated in an expectation-maximization (EM) algorithm^58^, until convergence is reached and a final estimate of the source locations is obtained (Fig. 4e). In a simulated environment, the actual numbers and locations of the source fluorophores are known, and can be compared to the final estimate. After matching estimates and actual locations using the Hungarian method^59^ assuming a known number of dyes (Fig. 4f), the absolute average estimation error can be calculated. The estimation errors in the *x*, *y* and *z* dimensions equal ± 21 nm (see Supplementary Table 11), thus the actual fluorophore locations can be approximated with high accuracy. A more detailed explanation and mathematical derivation of the algorithm can be found in the Supplementary Methods.

### Spatial distribution of functional groups

**Fig. 5:**
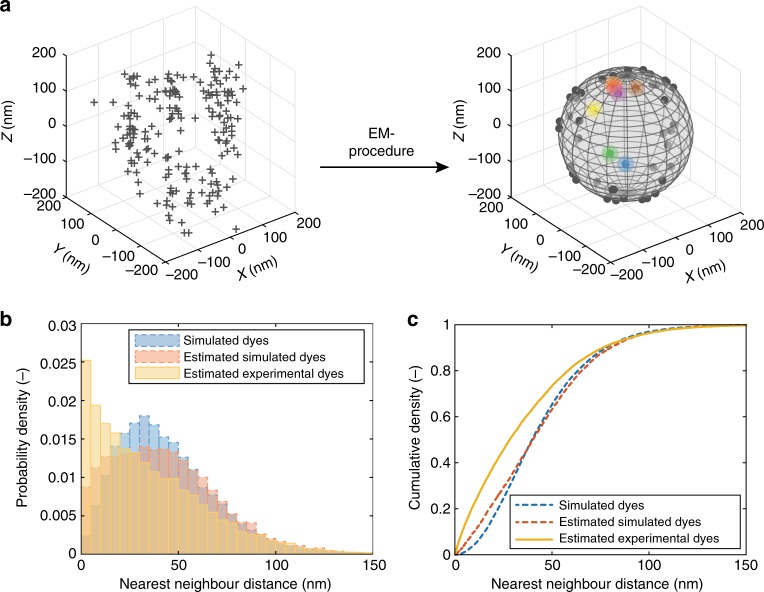
Analysis of the functional group distribution on the surface of nanoparticles. **a** 3D-dSTORM image of a multifunctional nanoparticle is analysed using an EM routine, to obtain the MLE estimate of the functional group locations (solid points). Transparent circles indicate the average estimation error. **b** Probability density function of NNDs. Plot includes NND distributions for the actual fluorophore locations in a stochastically homogeneous simulation (dashed blue, $N_{{\mathrm{NND}},{\mathrm{sim}}} = 4.2 \times 10^4$), for the estimated locations in a stochastically homogeneous simulation (dashed red, $N_{{\mathrm{NND}},{\mathrm{est}}} = 4.2 \times 10^4$) and the estimated locations in the experimental dSTORM measurement (solid yellow, $N_{{\mathrm{NND}},{\mathrm{exp}}} = 1.8 \times 10^4$). **c** Cumulative probability distribution of NNDs. Distribution analysis of low- and high-density beads are presented as Supplementary Figs. 14 and 16

To elucidate the functional group distribution on individual nanoparticles, Alexa647-appended particles have been investigated using the EM-estimation algorithm (Fig. 5a). The most common assumption for the functionalization process is for the fluorophore molecules to attach “fully randomly”, i.e. independently of other dyes or particle orientation. It is necessary to stress that even for fully random functionalization, stochastic variability in functional group densities along the bead is to be expected. This stochastic clustering can be conveniently quantified using the distribution of nearest-neighbour distances (NNDs)^58^. When the likelihood for a fluorophore to attach is constant over the surface of a nanoparticle, and thus independent of other bound dyes, the functionalization procedure can be represented as a homogeneous negative binomial point process. Thus, we have simulated a large population of beads using this “stochastically homogeneous” (from here on termed “homogeneous” for clarity) process at the appropriate average functionalization density, and have calculated the NND distribution of the functional groups (Fig. 5b, blue bars). The localization lists from these simulated beads have been subjected to the EM-algorithm, and the NND distribution for the location estimates has also been calculated (Fig. 5b, red bars). Comparison of these two distributions shows some flattening of the features below ±50 nm (the residual effect of overcounting), but the main characteristics of the NND distribution are preserved. This confirms property (iii), enabling us to draw valid inference on the NND distribution of the experimental beads.

Compared to the estimation based on the simulated dye distribution, the NND distribution for fluorophore locations obtained from the experimental images using the EM-algorithm (Fig. 5b, yellow bars) is rather different, also clearly visible in the cumulative distribution plots (Fig. 5c). One can already qualitatively conclude that the experimental data are more clustered (smaller NND) than would be expected when assuming homogeneity in the functionalization. In order to quantitatively confirm the observed deviation from homogeneity at the population level, we have performed statistical significance testing of the average NND per nanoparticle. Based on the estimated dye positions of a population of particles simulated under the homogeneity assumption (*N*_beads,sim/est_ = 937), we have constructed an empirical distribution of the average NND per bead. For each experimentally observed particle (*N*_beads,exp_ = 379), we have calculated the average NND of its estimated dye locations. These values are compared to the 2.5% and 97.5% quantiles of the estimated simulated (homogeneous) average NND distribution, which correspond to values that are very rare under the homogeneity assumption, and the number of observed values outside these boundaries is counted. If the homogeneous model describes the data accurately, this sum is ${\mathrm{Binomial}}(n = 379,p = 0.05)$ distributed. Using *α* = 0.05, the threshold value for accepting homogeneity is 26 observed values outside the specified boundaries. Experimentally, we find a value of 82 (*P* < 0.001, tailored test, see Supplementary Table 13), leading to the rejection of homogeneity in our experimental population. The low average NND found among these outliers indicates the presence of pronounced fluctuations in functional group density, resulting in a higher degree of clustering than the stochastic clustering that might be expected for a homogeneously functionalized bead.

### Effects of heterogeneity

**Fig. 6:**
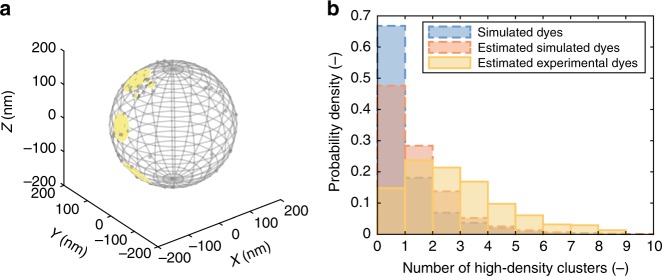
Occurrence of regions with a high functional group density. **a** Identification of high-density regions on the surface of a nanoparticle. High-density regions  (yellow circles) are defined as containing the estimated locations of ≥5 fluorophores (grey points) in a surface region with radius ≤50 nm. **b** Probability mass function of the number of high-density clusters on a nanoparticle. The plot depicts distributions for the (stochastically homogeneously) simulated fluorophore locations (dashed blue, $N_{{\mathrm{beads}},{\mathrm{sim}}} = 937$), the (stochastically homogeneously) simulated estimated fluorophore locations (dashed red, $N_{{\mathrm{beads}},{\mathrm{est}}} = 937$) and the experimentally observed nanoparticles (solid yellow, $N_{{\mathrm{beads}},{\mathrm{exp}}} = 379$)

In addition to the population-level analysis, dSTORM provides insight into the particle-to-particle variability of functional group distributions (Supplementary Figure 13). Since functionalization of nanoparticles does not take place homogeneously over the surface, finding a closed expression for the prediction of local functional group density is problematic. The exact form of the heterogeneity is unknown, may be variable with nanoparticle composition, size or functionalization chemistry, and is difficult to determine experimentally. Rather, a dSTORM-based approach can be applied to estimate fluorophore locations and empirically determine their arrangement, capturing bead-to-bead stochastic variability in the number of functional groups and their spatial distribution. As an example relevant to multivalent interactions, we quantify the presence of high-density regions on the surface of our experimental nanoparticles. Such a patch might elicit a biochemical response in a therapeutic setting or might allow displacement in competitive interactions^7,41^. Moreover, patchy particles may yield emergent properties and display interesting colloidal behaviour^60,61^. As an arbitrary threshold, we define a high-density region as the presence of five functional groups within a 50 nm arclength radius (Fig. 6a). The occurrence of these patches on the experimental nanoparticles has been quantified using our MLE procedure, showing a quantitatively different distribution and a higher average number of patches compared to the homogeneous case (Fig. 6b). Notice that the distribution for the simulated dyes (blue) illustrates the enhanced stochastic clustering of the real beads compared to their simulated counterparts. Let us assume that the presence of at least three patches results in an effective interaction. Now, based on the estimations, the probability that an arbitrary bead cannot effectively interact, $P(N_{{\mathrm{clusters}}} < 3)$, equals 0.90 for the (homogeneously) simulated beads (which is close to the probability of 0.91 underlying the simulations) and 0.60 for the experimental beads (Fig. 6b). As a consequence, on average, more than 28 homogeneously functionalized beads are required to achieve 95% probability of successful signalling $\left( {1 - 0.9^{28} = 0.948} \right)$, as opposed to only 6 based on the empirical distribution $\left( {1 - 0.6^6 = 0.953} \right)$. When more patches are needed for effective interaction, this difference is exacerbated. Hence, these results show that the multivalent action of nanoparticles prepared through such covalent functionalization cannot be predicted correctly by assuming homogeneously reacted species, and efficacy will be likewise affected.

## Discussion

An approach combining super-resolution microscopy with stochastic modelling and statistical estimation has been outlined, in which we demonstrate improved interpretation of nanoparticle surface functionality. We have verified its ability to capture the dye photophysics (i), incorporate functional group quantities (ii) and reconstruct spatial patterns (iii), and have thus proven the accurate estimation of fluorophore locations on the nanoscale. Functionalization of nanostructures is generally performed with the (implicit) aim of creating a uniform functional group pattern, but we have identified various sources of variability when applying common protocols. Stochastic fluctuations proved insufficient to explain the experimentally observed variability in dye-labelled polystyrene nanoparticles, and the common hypothesis of stochastically homogeneous (“fully random”) attachment of functionalities was rejected.

The large particle-to-particle variation in the number of functional groups and the clear heterogeneity of their distribution on the nanoparticle surface imply some measure of association between attachment events. This heterogeneity may be related to the distribution of reactive sites on the beads, to the functionalization chemistry or other factors. While its exact origin is currently unclear, it is likely prevalent in synthetic nanoparticles customized for various applications, taking into account the ubiquity and robustness of the functionalization procedures applied here. Considering the growing relevance of controlling the surface topology of functional nanoparticles in both materials science and biomedical developments^62,63^, elucidation of the cause and form of the heterogeneity are priorities for future research.

Of course, inhomogeneous distributions of moieties over surfaces and volumes are consequential in a wider range of artificial, as well as biological, nanosystems. The MLE based analysis of such distributions can be generalized for different probes, densities and scales, although the requirements for the algorithm may be different and hence call for optimization. Validation may be performed in silico as demonstrated here or might be accomplished using discrete reference structures with well-defined fluorophore locations (e.g. DNA origami^47^). Especially when the underlying molecular distribution is inaccessible, our approach is suitable for measuring probe locations and separation. In this way, it might function similarly to other clustering methods, and benchmarking relative performance and location precision might be worthwhile.

Variation in surface functionality affects multivalent interactions in therapeutic, nanotechnological and biochemical applications. It is crucial to be aware of the difference between average functionalization densities and the real, highly variable, coverage of individual nanoparticles. Controlling and harnessing this interparticle variability requires single-particle analysis of functional group densities, of which the dSTORM-based empirical analysis of high-valency regions on synthetic nanoparticles described in this work is an example. We expect that improved accessibility of such approaches will contribute to significant improvements in the design of novel functional nanostructures.

## Methods

### Nanoparticle labelling

Carboxylic acid functionalized polystyrene beads (Spherotech CP-025, composition: polysterene (amorphous), functionality: carboxyl, functional group content: 50 mol g^−1^ solid, diameter: 337 nm, zeta potential: −63.8 mV) were suspended at 0.25% w/v in phosphate-buffered saline (pH 7.2) followed by addition of 10 eq. EDC (Sigma-Aldrich, 240 μg), 25 eq. NHS (Sigma-Aldrich, 357 μg) and Alexa647-Cadaverine (Invitrogen). Single-dye beads were created using 10^−6^ eq. of Alexa647-Cadaverine (0.2 ng, added through a dilution series in MilliQ). Multifunctional beads of different densities were created using 10^−4^ (L), 10^−3^ (M), 10^−2^ (H) and 10^−1^ (VH) eq. of Alexa647-Cadaverine, respectively. Reactions were shaken at room temperature for 4 h. Subsequently, the beads were iteratively centrifuged and resuspended in MilliQ (three iterations), followed by extensive dialysis for 24 h (1 kDa MWCO; Spectrum Labs, three iterations).

### dSTORM imaging

In order to perform dSTORM imaging, sample chambers were created as follows. Glass microscope coverslips (No. 1.0, 26 ×22 mm, thickness 0.15 mm) were consecutively immersed in acetone, isopropanol and MilliQ and sonicated for 10 min in each solvent. Next, a fresh solution of Piranha etch was prepared (3:1 v/v concentrated H_2_SO_4_:H_2_O_2_ (aq, 30%)) in which the slides were incubated for 15 min. Finally, the slides were washed thoroughly with MilliQ and acetone before drying under N_2_-flow. In order to introduce fiducial markers, TetraSpeck™ Microspheres (solution) were diluted 50× in ethanol, and 5 μL of this solution was placed on the coverslip. After the solution had spread over the entire surface, the ethanol was evaporated under N_2_-flow. Subsequently, an imaging chamber was constructed using a glass slide, two strips of double-sided tape and the coverslip. The imaging chamber was incubated with 0.1% poly-l-lysine solution (Sigma-Aldrich) for 10 min, flushed three times with MilliQ and then incubated with a suspension of Alexa647-functional nanoparticles for 30 min. After this incubation, the imaging chamber was flushed with MilliQ (three times) and dSTORM buffer (three times). dSTORM buffer contains 50 mM Tris-HCl (pH 8.0), 10 mM NaCl, 5% w/v glucose, 0.1 M MEA (mercaptoethanolamine; Sigma-Aldrich) and an oxygen scavenging system (0.5 mg per mL glucose oxidase, 40 μg per m catalase). dSTORM images were acquired using a Nikon N-STORM 4.0 system configured for total internal reflection fluorescence imaging. Alexa647-labelled nanoparticles were illuminated using a 647 nm laser; the fiducial markers were illuminated using a 488 nm laser. No UV activation was employed. Fluorescence was captured using a Nikon ×100, 1.4 NA oil immersion objective and passed through a quad-band pass dichroic filter (97335, Nikon). Images were acquired using a 256 × 256 pixel region of interest (pixel size 160 nm) on a CMOS camera. For a measurement, 50,000 frames were acquired in the 647 channel at maximum camera speed (frametime ±4 ms for this ROI on the CMOS camera). Sample imaging was alternated every 100 frames by a single 488 channel frame for drift correction. Three-dimensional acquisition was performed using the astigmatic method, calibrated by Z-stepping a sample with a high-density of fiducial markers at optimized laser intensity. dSTORM images were analysed using the dSTORM module of the NIS Elements software (Nikon). Preprocessing of the acquired data is described in the Supplementary Methods.

### Probabilistic model and data simulation

The probabilistic model for the dye dynamics describes the blinking behaviour (Supplementary Figs. 2 and 3), the number of localizations per bead (Supplementary Figs. 4–6) and the observation of a signal given its source (Supplementary Eqs. 4–43 and Supplementary Figs. 7–9). The details and reasoning behind the model are presented in the Supplementary Methods. Based on this probabilistic model computer simulated beads have been generated under the assumption of homogeneous functionalization.

### Estimation of dye locations

Based on the probabilistic model the number of dyes can be estimated from a collection of localizations (Supplementary Eqs. 44–46). Subsequently, an EM-algorithm is used to estimate their positions on the bead (Supplementary Eqs. 47–63). The details and reasoning behind the estimation procedure are presented in the Supplementary Methods.

### Distribution analysis

The processed dSTORM images are compared with the processed simulated localization sets to ascertain homogeneous functionalization. Inference is drawn based on the average NND per bead using a modified randomization test (Supplementary Eqs. 64–67 and Supplementary Figs. 14–16). The details and reasoning behind this distribution analysis are presented in the Supplementary Methods.

## Supplementary information

Supplementary Information

## Data Availability

The data that support the findings of this study are available from the corresponding author upon reasonable request.

## Code availability

The R code of the EM-algorithm is available from the corresponding author on reasonable request.
