# Supplementary material for: A stochastic view on surface inhomogeneity of nanoparticles
Source: Nat Commun. 2019 Apr 10;10:1663. doi: 10.1038/s41467-019-09595-y (PMC6458121; doi:10.1038/s41467-019-09595-y)
Supplement: Supplementary file 1 — Supplementary Information [file 41467_2019_9595_MOESM1_ESM.pdf]

Supplementary Information

A stochastic view on surface inhomogeneity of nanoparticles

Post et al.

Contents

Supplementary Methods **2**
Data processing . . . 2
Statistical model & Data analysis . . . 3
Emitters on single dye beads . . . 3
Functionalized beads . . . 6
Number of localizations . . . 6
Spatial patterns of single localizations . . . 10
Lateral coordinates of the localization . . . 10
Astigmatism to estimate axial coordinate of dye location . . . 10
Estimation errors . . . 12
Lateral uncertainty . . . 14
Axial uncertainty . . . 16
Uncertainty in width estimates . . . 16
Mixed Normal distribution . . . 18
Estimators for the number of dyes . . . 22
Estimate for the real position of the dyes . . . 24
Mathematical theory . . . 24
Initial positions emitters . . . 27
Performance algorithm . . . 27
Distribution analysis . . . 30
Bead-specific clustering quantification . . . 30
Goodness of fit test . . . 30
Practical application of the clustering quantification . . . 34

Supplementary Note 1 **36**

Supplementary Note 2 **36**
Poisson point process . . . 36
Markov processes . . . 37

Supplementary References **38**

Supplementary Methods

Data processing

dSTORM datasets as exported by NIS Elements comprise lists of localizations and their corresponding properties. In order to allow for a quantitative analysis, these datasets have been pre-treated using Matlab (scripts available on request). In brief, in this pre-treatment, the list of localizations was imported into Matlab and fiducial marker-related as well as Z-rejected localizations were removed from the imaging channel (a thorough correlation analysis was performed to confirm that no difference in any photophysical parameters existed between Z-rejected and accepted localizations). Subsequently, drift correction was performed using the fiducial markers and all localizations acquired after reaching blinking equilibrium (set at frame $10,000$ as determined by completion of a fast decay in number of localizations per frame) were collected. In this dataset, individual beads were isolated using a density-based clustering algorithm (selecting clusters of more than 2/5 localizations in a 80/225 nm range, for monofunctional and multifunctional beads respectively). After excluding erroneous clusters (based on the criteria size-radius between 125 and 180 nm - and number of localizations - 10/90/250/450 for L/M/H/VH densities), the localizations datasets were exported for further distribution analysis in R.

Subsequently, the datasets are further processed in R before analysis. First, localizations based on 300 or less photons and those that are based on more than 4000 photons were removed. Based on this criterion 234 (SDB), 191(L), 630 (M), 2318(H) and 8234 (VH) localizations were removed. Second, we have removed various entire clusters (beads) that contain an unrealistic number of localizations. The threshold values (the rounded 99% quantiles of the data) were 45 (SDB), 150 (L), 550 (M) and. 2500 (H). For the VH dataset we used a lower bound instead, the 1% quantile 1750. As a result, 27 out of 2233 (SDB), 7 out of 281 (L), 1 out of 380 (M), 2 out of 226 (H) and 6 out of 412 (VH) beads were removed. The number of observations (n), the mean, the standard deviation (SD), median, 25% quantile (Q25) and the 75% quantile (Q75) of the number of localizations and the number of Photons are presented in Supplementary Table 1. In this work the data from the VH beads are solely used for fitting the photon count distribution.

Variable	Density	n	Mean	SD	Median	Q25	Q75
Number of	SD	2206	7.65	7.00	5.00	3.00	9.00
localizations	L	274	36.0	25.0	27.0	19.0	49.0
	M	379	228.5	89.2	219.5	153.5	284.5
	H	224	1008.1	398.2	986.0	672.5	1264.0
	VH	406	3622.1	862.1	3573.5	3086.0	4070.0
Photons	SD	16865	821	596	613	464	910
	L	10128	623	291	544	433	712
	M	868337	646	282	574	460	748
	H	229850	629	282	555	444	725
	VH	1470557	740	371	638	494	871

Supplementary Table 1: Summary statistics

In the dSTORM procedure, low as well as high values of e.g., localization counts are removed from the data, making it natural to work with conditional distributions. We do this throughout our analysis.

Statistical model & Data analysis

Random variables are written with a capital letter (e.g. X), and realizations of random variables with lower-case letters (e.g. x).

Emitters on single dye beads

First, a model for the photon emission dynamics of a dye on a bead containing solely this dye is presented. The parameters are estimated based on the SD dataset without suffering from overcounting. Next, the model is extended to a model for functionalized beads.

The behaviour of a single dye is modelled as a three-states Markov process. The three states represent a dark state (D), an active state (A), and a bleached state(B). This model has been used before to address the overcounting problem in PALM imaging, see [1]. During super-resolution acquisition, photoswitchable dyes are susceptible to several types of photophysical transitions, leading to various dark (i.e., non-emissive) states. Generally, these can be classified as either unintended, short-lived dark states (e.g., due to triplet transitions) [2, 3],or specifically engineered, long-lived dark states (e.g., due to photoswitching reagents or illumination) [4, 5]. These transitions are incorporated by extending the Markov model with an unknown amount of dark states. We single out a switching agent-induced dark state (D) whose time of visit is significantly longer than in the other dark states (D_1,D_2,...,D_n). Instead of an active state an emitting state (E), in which photons are generated, is defined such that $A = E \cup \{\bigcup_{i=1}^{n} D_i\}$. The state space and possible transitions of the Markov chain for the model are presented in Supplementary Figure 1. A dye is 'active' when it is in the emitting state or in one of the shorter dark states.

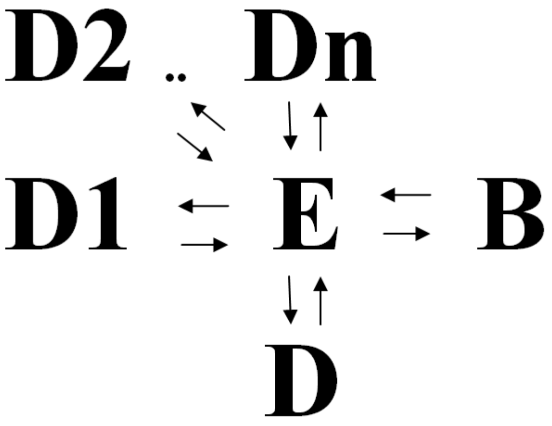

Supplementary Figure 1: Markov Chain model for blinking dynamics of a single dye

After being active, the dye will go back to state D or will be irreversibly photobleached (B). The probability of bleaching when making a transition from the emitting state E equals $p_{\text{bleach}} = \frac{k_b}{k_b + k_d}$, where k_a, k_b, and k_d are the transition rates. The bleached state is an absorbing state which guarantees that the Markov Process reaches its limiting distribution (all dyes bleached) eventually, see e.g. [24].

The total number of blinks of a specific single dye equals one plus the total number of successive visits to the dark state. The probability of returning to the dark state is constant and equals $1 - p_{\text{bleach}}$. To improve readability p_{bleach} is referred to as $\tilde{p}$. The total number of blinks (B) is modelled with a geometric distribution with success probability $\tilde{p}$, so $B \sim Geo(\tilde{p})$. It is difficult in practice to distinguish between signals of beads with only one dye attached and background noise. Therefore, only data from dyes that blinked at least three times is used. Furthermore, the estimate is based on dyes that blink at most 25 times for reasons of robustness. Here the appropriate model is a conditioned geometric distribution. Let q be the observed number of blinks. Using the SD data a maximum likelihood estimation (MLE) estimate of $\hat{p}_{\text{bleach}} = 0.2084$ is derived. In Supplementary Figure **??** the histogram (binned per 2 blinks for clear visualization)of the observed (conditioned) blink data is shown. The green curve equals the probability density of the conditioned geometric distribution with success probability $\hat{p}_{\text{bleach}}$. In Supplementary Figure **??** the empirical cumulative distribution function (ECDF) of the data and the cumulative distribution function (CDF) of the conditional geometric distribution are presented.

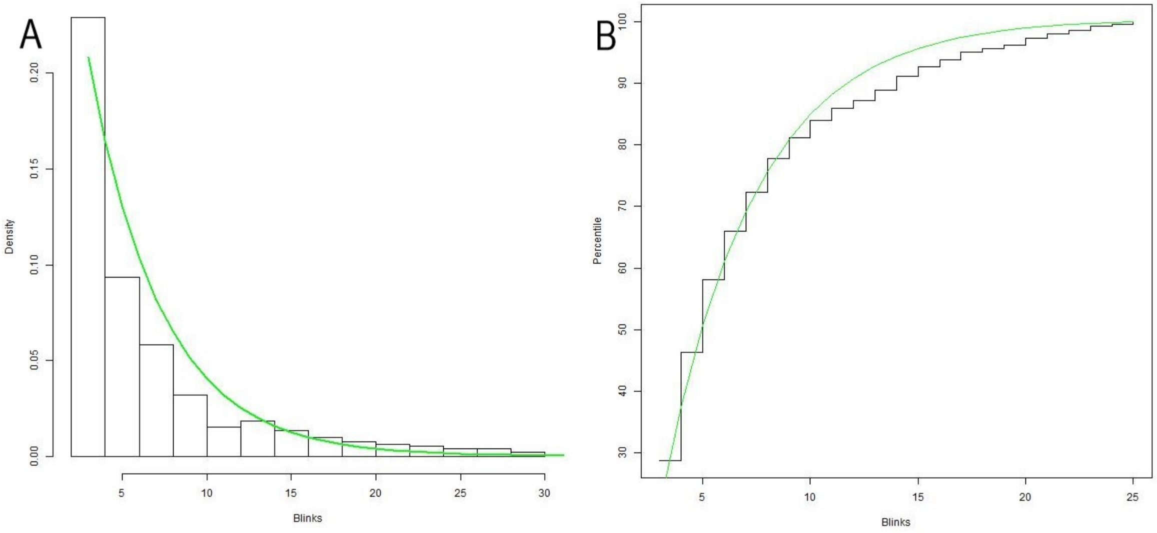

Supplementary Figure 2: Number of blinks distribution. a.) Histogram of observed number of blinks and geometric probability mass function (solid green). b.) ECDF of the observed number of blinks (solid black) and the geometric CDF (solid green).

Although the tail (number of dyes with large number of blinks) of the observed data deviates from the conditional geometric model, this model seems to describe the blinks count reasonably well for our modeling purpose.

The time that a dye spends in the active state consists of short periods when the dye is emitting photons alternated by short periods, D_1,D_2,..., D_n, without emission although the dye is still formally in an 'active' (i.e., not bound to switching agent) state. The lengths of the periods T_i in which the dye is emitting are assumed to be exponentially, $Exp(\cdot)$, distributed. The number N of the emission periods within an active period is unknown. As a result, the

total emission time, defined as T_{on}, equals

$$T_{\text{on}} = \sum_{i=1}^{N} T_i. \tag{1}$$

The number of photons released by an emitter can be assumed to be proportional to the time in the emissive state [3]. More specifically, $N_{\text{Photons}} = \nu \cdot T_{\text{on}}$, where ν is a numeric constant. This results in

$$N_{\text{Photons}} = \sum_{i=1}^{N} \nu \cdot T_i = \sum_{i=1}^{N} Y_i, \text{ where } Y_i \sim Exp(\lambda \cdot \nu^{-1}). \tag{2}$$

If we would assume N to be a deterministic value, then the continuous approximation of the number of photons would be a gamma distribution with shape parameter N and scale parameter $\nu \cdot \lambda^{-1}$, denoted by $Gamma(N, \nu \cdot \lambda^{-1})$. To prove this the moment generating function (MGF), which equals $\mathbb{E}[\mathrm{e}^{X \cdot t}]$, is used. The MGF uniquely defines the probability distribution and therefore equality in MGF implies equality in distribution, see e.g. [20, Chapter 5]. The MGF of a $Gamma(\omega, k)$ distribution equals $(1 - t\omega)^{-k}$. The MGF of the sum of interest equals

$$\begin{aligned}
\mathbb{E}[\mathrm{e}^{t \sum_{i=1}^{N} Y_i}] &= \mathbb{E}[\prod_{i=1}^{N} \mathrm{e}^{tY_i}], \\
&= \prod_{i=1}^{N} \mathbb{E}[e^{tY_i}], \\
&= \mathbb{E}[e^{tY_1}]^N, \\
&= (1 - \nu \cdot t \cdot \lambda^{-1})^{-N}.
\end{aligned} \tag{3}$$

When N is random this result is not true. However, if N has low variance the gamma distribution would be a good candidate to approximate the behaviour of interest. Therefore the fit of a gamma distribution with shape parameter k and scale parameter θ is investigated[1], where $\theta = \nu \cdot \lambda^{-1}$, to the data describing the number of photons emitted per blink. The parameters are estimated via a numerical optimization procedure of the likelihood.

Using the PROC NLMIXED available in the statistical software SAS we numerically optimize the conditional likelihood ($300 \leq n_{\text{Photons}} \leq 4000$) for the shape and the scale, here $\nu \cdot \lambda^{-1}$, parameters. The photon count data in all datasets (SD, L, M, H and VH) is used. Remarkably, the fitting $\tilde{N}_{\text{Photons}} = N_{\text{Photons}} - 300$, with a gamma distribution seems more appropriate than fitting the original data with the conditional distribution. Therefore, $N_{\text{Photons}} - 300$ is modelled for which $\hat{k} = 1.6168$ and $\hat{\theta} = 0.00384$ are found. The histogram of the data (binned by 100 photons), the gamma probability density are plotted in Supplementary Figure **??**. Moreover, the ECDF and the gamma CDF are shown in Supplementary Figure **??**.

[1]We parametrize the gamma distribution as $f(x; k; \theta) = x^{k-1} \frac{\exp(-x/\omega)}{\omega^k \Gamma(k)}$

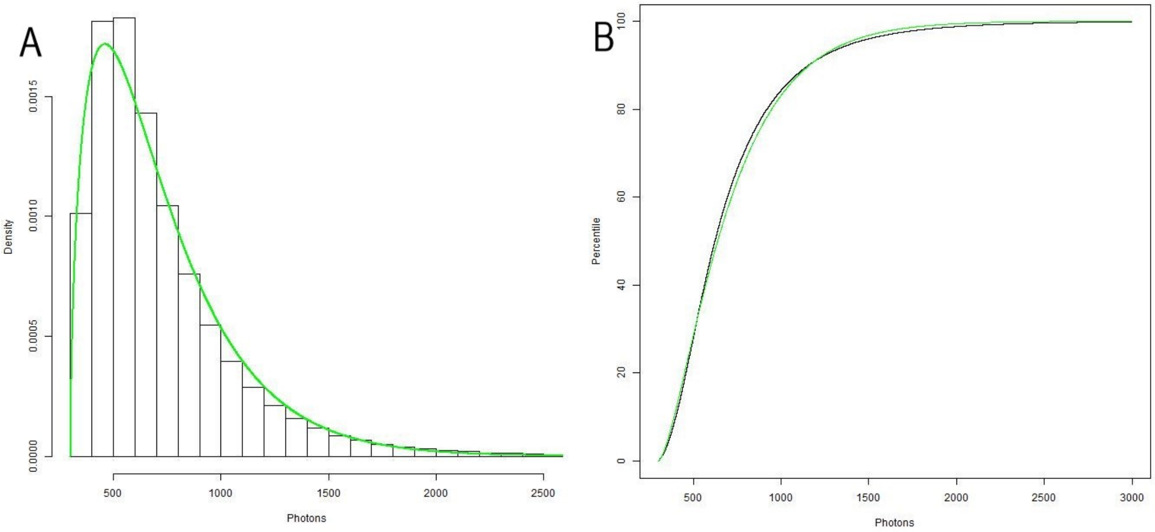

Supplementary Figure 3: Number of photons released per blink distribution. a.) Histogram of the observed number of photons and the gamma probability density (solid green). b.) ECDF of the observed number of photons (solid black) and the gamma CDF (solid green).

Functionalized beads

The null model for the spatial pattern of the dyes is a homogeneous Poisson point process (PPP) on the surface of the nanoparticle which is a sphere, see Supplementary Note 2. Such a homogeneity assumption is often made in the literature, see e.g. [7]. This homogeneity is a crucial assumption that we aim to investigate. Furthermore, the different dyes are assumed to operate independently of each other.

Number of localizations

Our goal is to estimate the number of dyes on a given bead. We observe the number of localizations and since the number of blinks per dye is random, the number of dyes on the bead is unknown. Therefore, it is difficult to estimate the parameter of the Poisson distribution describing the number of dyes. However, an appropriate model for the number of localizations per dye and data of the total number of localizations per bead are available. Since the L, M and H densities of functionalization are expected to yield the most interesting results from a multivalency point of view, these will be treated in further analysis.

Let B_i be the number of localizations originating from dye i. As a result of the data acquisition process, we have that $b_{\mathrm{L}} \leq B_i$ with different b_{L} which are presented in Supplementary Table 2. The conditioned compound Poisson process is defined as

$$Q_j = \sum_{i=1}^{X_j} B_i. \tag{4}$$

Here Q_j equals the total number of blinks of bead j and X_j equals the number of non-bleached emitters at equilibrium-time on the bead. The term *compound* is used when summing a random number, here X_j, of random variables. The number of dyes that are not

bleached when the equilibrium is reached is distributed as a binomial random variable, $X_j \sim Bin(X_{\text{start}}, 1 - p_{\text{bleach}})$. Here X_{start} represents the total number of dyes on a bead and p_{bleach} is the probability that a dye is in the bleached state when the equilibrium is reached. Since X_{start} is given by a Poisson random variable, X_j also follows a Poisson distribution, see Appendix . Here we focus on the point process when the process has reached the equilibrium. The probability distribution of the conditional compound Poisson distribution, stated in Supplementary Equation 4, is implemented in SAS. Next, the Poisson parameter estimate $\hat{\Lambda}(S)$ that optimizes the likelihood for the different bead densities is computed. However, as shown in Supplementary Table 1, the variance in the localizations count data is much larger than the expected value. Since for a Poisson random variable, the mean is equal to the variance, the assumption of Poisson counts is not appropriate. The additional variation that is not explained by the model is often referred to as 'overdispersion'. In the case of a Poisson random variable it is common to propose a Poisson mixture model, the Poisson parameter itself is a random variable, for example a Poisson-Gamma mixture distribution. This Poisson-Gamma mixture distribution is equivalent to a Negative Binomial, $NB(r, p)$, distribution. The conditional compound NB probability distribution is implemented in SAS and a MLE estimate for all bead densities is derived. The MLE estimates for the Poisson parameter, $\hat{\Lambda}(S)$, and the NB parameters, $\hat{r}$ and $\hat{p}$, are presented in Supplementary Table 2. In addition, the graphical representations are presented as Supplementary Figures 4-6. Here, the

Density	b_{L}	$\hat{\Lambda}(S)$	$\hat{r}$	$\hat{p}$
L	10	7.78	1.71	0.17
M	90	46.82	6.54	0.12
H	250	202.28	6.17	0.03

Supplementary Table 2: MLE estimates of the parameters of the number of localizations distribution for both the $Poi(\hat{\Lambda}(S))$ and the $NB(\hat{r}, \hat{p})$ distributions.

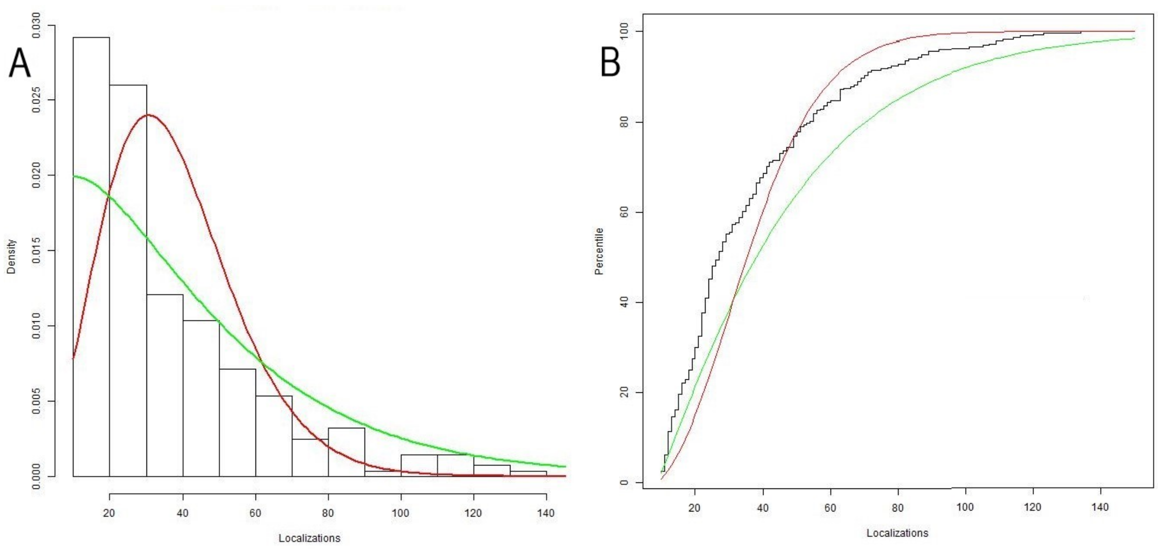

Supplementary Figure 4: Low-density beads number of localizations distribution. a). Histogram of the observed number of localizations (binned by 20 counts) and probability mass functions of the Poisson (solid red) and Negative Binomial distributions (solid green). b). ECDF of the observed number of localizations per bead and the CDFs of the Poisson (solid red) and Negative Binomial distributions (solid green).

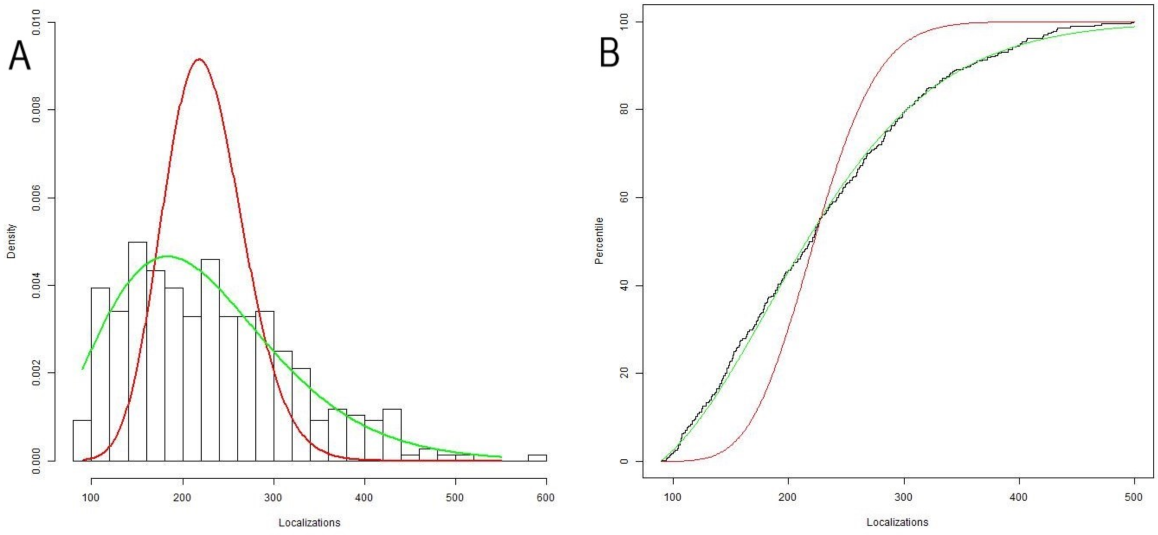

Supplementary Figure 5: Medium-density beads number of localizations distribution. a). Histogram of the observed number of localizations (binned by 20 counts) and probability mass functions of the Poisson (solid red) and Negative Binomial distributions (solid green). b). ECDF of the observed number of localizations per bead and the CDFs of the Poisson (solid red) and Negative Binomial distributions (solid green).

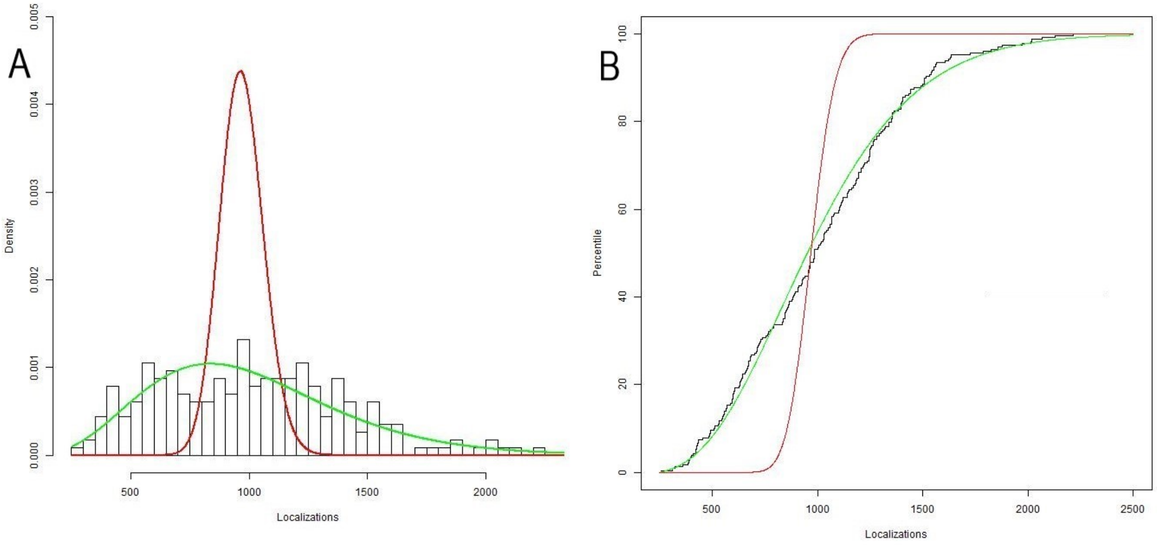

Supplementary Figure 6: High-density beads number of localizations distribution. a). Histogram of the observed number of localizations (binned by 50 counts) and probability mass functions of the Poisson (solid red) and Negative Binomial distributions (solid green). b). ECDF of the observed number of localizations per bead and the CDFs of the Poisson (solid red) and Negative Binomial distributions (solid green).

For medium- and high-density beads, the improved fit quality of the compound negative binomial distribution can be clearly seen in the cumulative distribution functions, demonstrating larger variability than expected by traditional count statistics. This overdispersion may indicate dependence between the reactions of different fluorophore molecules on a particular bead or dispersity at the bead level, thus casting doubt on the assumption of fully random functionalization that is almost universally adopted in the field.

Spatial patterns of single localizations

The basic principles for obtaining position estimates of single dyes as implemented in the N-STORM setup, which serve as a starting point for the procedures described in this subsection, are publicly available [10, 11]. These publications have been supplemented and clarified in personal communication with Nikon Instruments Europe B.V.

Entering the photobleached state (B) is an irreversible process i.e., the dye cannot leave this state. If the dye enters the dark state (D) it will return to the active state (A) after an exponential time. In this ON-state (A), an emitter is activated and starts emitting photons. Based on these, the position of the dye is estimated.

The position of the jth localization, $\boldsymbol{L}_j = (x, y, z)$ is modelled as a realization of a multivariate normal distribution centered at the exact position of the emitter $\boldsymbol{\mu}_j$ and with diagonal covariance matrix. The elements on the diagonal are the theoretical variances $\boldsymbol{L}_j \sim \mathcal{N}\left(\boldsymbol{\mu}_j, \mathrm{i}\cdot\boldsymbol{\sigma}_j\right)$, where $\boldsymbol{\sigma}_j^2 = \left(\sigma^2_{\mathrm{x},j}, \sigma^2_{\mathrm{y},j}, \sigma^2_{\mathrm{z},j}\right)$ and I the 3×3 identity matrix.

Lateral coordinates of the localization

The photons emitted are captured by a camera and a two-dimensional diffraction limited spot is observed. Next, the Nikon software fits a 2D-Gaussian distribution to this data using the method of least squares. This procedure gives a Gaussian Mask Estimator (GME) for the lateral coordinate, (x, y), of the dye and an estimate for the variance in both x- and y-direction [22]. Let N_{photons} equal the total number of photons captured during one active state, and let $i$represent a specific pixel. Then, the expected number of photon counts in pixel $i$is given by

$$E_i(\mu_\mathrm{x}, \mu_\mathrm{y}, \sigma^2_\mathrm{x}, \sigma^2_\mathrm{y}) = N_{\text{photons}} \cdot p_{i,\mu_\mathrm{x},\mu_\mathrm{y},\sigma^2_\mathrm{x},\sigma^2_\mathrm{y}} + b^2, \tag{5}$$

where $p_{i,\mu_\mathrm{x},\mu_\mathrm{y},\sigma^2_\mathrm{x},\sigma^2_\mathrm{y}}$ equals the probability that a photon is observed in pixel i based on the Gaussian distribution with the corresponding parameters and b^2 the expected number of photons from the background signal [22]. Let n_i equal the realized photon count in pixel i. Then the GME of the location and variability parameters minimizes

$$\chi^2(\mu_\mathrm{x}, \mu_\mathrm{y}, \sigma^2_\mathrm{x}, \sigma^2_\mathrm{y}) = \sum_i \left(n_i - E_i(\mu_\mathrm{x}, \mu_\mathrm{y}, \sigma^2_\mathrm{x}, \sigma^2_\mathrm{y})\right)^2. \tag{6}$$

Astigmatism to estimate axial coordinate of dye location

The axial location of an emitter is estimated with different techniques, see [8]. The dSTORM software applies the astigmatism method, which is known to be the most cost-effective technique, see [9]. Astigmatism is introduced using a weak cylindrical lens, and can be used to obtain resolution in the axial direction. w_x and w_y are defined as the widths, in the x- and y-direction, of the diffraction limited spot and equal two times the standard deviation of the assumed underlying Gaussian distribution. Due to the cylindrical lens, the focal planes for the x and y directions are different ($w_\mathrm{x} \neq w_\mathrm{y}$). As a result the captured image is different for emitters at different z positions, see [11]. A dye that is active in the average focal plane, where $z = 0$, results in a round pattern, while an emitter with a higher z position results in a more ellipsoidal image (long axis along x direction). The same effect on the image would occur for a lower z position (this time the long axis along the y direction), see [13]. Huang et al.

[11] experimentally generate a calibration curve to determine the z position based on w_x and w_y. In our experiments, a similar calibration curve was obtained by imaging a slide covered with fiducial markers in high-density. The stage piezo is used to move the slide around the focal plane in well-defined steps, and the shape of the diffraction-limited spots is captured as a function of the axial position. The NIS Elements software [12] subsequently fits the widths of the spots in x- and y-dimensions using the following algebraic functions

$$w_x(z) = w_{0,x} \cdot \sqrt{1 + \left(\frac{z-g_x}{Z_{r,x}}\right)^2 + A_x \cdot \left(\frac{z-g_x}{Z_{r,x}}\right)^3 + B_x \cdot \left(\frac{z-g_x}{Z_{r,x}}\right)^4}, \tag{7}$$

$$w_y(z) = w_{0,y} \cdot \sqrt{1 + \left(\frac{z-g_y}{Z_{r,y}}\right)^2 + A_y \cdot \left(\frac{z-g_y}{Z_{r,y}}\right)^3 + B_y \cdot \left(\frac{z-g_y}{Z_{r,y}}\right)^4}. \tag{8}$$

Here, w_0 is the image width for a dye in the focal plane (PSF), g is the offset of the x or y focal plane from the average focal plane (astigmatism), $Z_{r,x}$ and $Z_{r,y}$ are the focal depth of the microscope in the x- and y-dimensions respectively. A and B are polynomial coefficients to compensate for optical aberrations. The parameter values of the calibration curve used in our analysis are presented in Supplementary Table 3.

parameter	$w_{0,x}$	$w_{0,y}$	g_x	g_y	$Z_{r,x}$	$Z_{r,y}$	A_x	A_y	B_x	B_y
value	289.92	268.35	212.0	294.06	510.44	490.06	1.2155	−0.248	−0.669	0.0169

Supplementary Table 3: Parameters calibration curve

To estimate the axial dye coordinate z, the widths in both lateral directions are estimated as

$$\hat{w}_x = 2\sqrt{\hat{\sigma}_x^2} = 2\sqrt{\frac{1}{N_{\text{photons}}} \sum_{i=1}^{N_{\text{photons}}} (x_{x,i} - \hat{\mu}_x)^2}, \tag{9}$$

$$\hat{w}_y = 2\sqrt{\hat{\sigma}_y^2} = 2\sqrt{\frac{1}{N_{\text{photons}}} \sum_{i=1}^{N_{\text{photons}}} (x_{y,i} - \hat{\mu}_y)^2}. \tag{10}$$

Next, the estimated widths $(\sqrt{\hat{w}_x}, \sqrt{\hat{w}_y})$ are projected on the parametric curve defined in Supplementary Equation 7 and Supplementary Equation 8. This projection is referred to as (a, b). If $\sqrt{(\hat{w}_x - a^2)^2 + (\hat{w}_y - b^2)^2}$ i.e., the absolute distance of the projection, is too large, then the shape of the PSF is not correctly captured by the calibration curve. In this case, NIS Elements assumes the localization to be of low quality and discards it ('Z-rejection'). In a broad data analysis, no correlation of Z-rejection to any measured photophysical property could be found. Hence, this process is modelled to occur with a set (constant) probability for any captured localization. For non-rejected localizations, $\hat{\mu}_z$, the estimate for the height of the dye, satisfies

$$\hat{\mu}_z = w_x^{-1}(z)(a^2) = w_y^{-1}(z)(b^2). \tag{11}$$

Since the point (a^2, b^2) lies on the calibration curve, the projection guarantees the equality in Supplementary Equation 11.

Estimation errors

Mortenson, Churchman, Spudich and Flyvbjerg [22] approximate the variance of the GMEs. Using the notation introduced in Subsubsection , they show that, for $\hat{\theta} = \hat{\mu}_{\mathrm{x}}, \hat{\mu}_{\mathrm{y}}, \hat{\sigma}^2_{\mathrm{x}}, \hat{\sigma}^2_{\mathrm{y}}$, and with $\sum_i$ the sum over all pixels as in the previous subsection,

$$\mathrm{var}\left(\hat{\theta}\right) = \frac{\sum_i \left(p_{i,\hat{\mu}_{\mathrm{x}},\hat{\mu}_{\mathrm{y}},\hat{\sigma}^2_{\mathrm{x}},\hat{\sigma}^2_{\mathrm{y}}} + \frac{b^2}{N_{\mathrm{Photons}}}\right)\left(\frac{\partial p_{i,\hat{\mu}_{\mathrm{x}},\hat{\mu}_{\mathrm{y}},\hat{\sigma}^2_{\mathrm{x}},\hat{\sigma}^2_{\mathrm{y}}}}{\partial \theta}\right)^2}{N_{\mathrm{Photons}}\left(\sum_i \left(\frac{\partial p_{i,\hat{\mu}_{\mathrm{x}},\hat{\mu}_{\mathrm{y}},\hat{\sigma}^2_{\mathrm{x}},\hat{\sigma}^2_{\mathrm{y}}}}{\partial \theta}\right)^2\right)^2}. \tag{12}$$

For sake of saving space let $p_i(x_i, y_i) \equiv p_{i,\hat{\mu}_{\mathrm{x}},\hat{\mu}_{\mathrm{y}},\hat{\sigma}^2_{\mathrm{x}},\hat{\sigma}^2_{\mathrm{y}}}$. If $p = \frac{1}{2\pi\cdot\sigma_{\mathrm{x}}\cdot\sigma_{\mathrm{y}}}\exp\left(-\frac{(x-\mu_{\mathrm{x}})^2}{2\sigma^2_{\mathrm{x}}} - \frac{(x-\mu_{\mathrm{y}})^2}{2\sigma^2_{\mathrm{y}}}\right)$, then the next identities hold:

$$\int p^3(x-\mu_{\mathrm{x}})^4 dxdy = \frac{\sigma^2_{\mathrm{x}}}{36\pi^2\cdot\sigma^2_{\mathrm{y}}}, \tag{13}$$

$$\int p^3(x-\mu_{\mathrm{x}})^2 dxdy = \frac{1}{36\pi^2\cdot\sigma^2_{\mathrm{y}}}, \tag{14}$$

$$\int p^3 dxdy = \frac{1}{12\pi^2\cdot\sigma^2_{\mathrm{x}}\cdot\sigma^2_{\mathrm{y}}}, \tag{15}$$

$$\int p^2(x-\mu_{\mathrm{x}})^4 dxdy = \frac{3\sigma^3_{\mathrm{x}}}{16\pi\sigma_{\mathrm{y}}}, \tag{16}$$

$$\int p^2(x-\mu_{\mathrm{x}})^2 dxdy = \frac{\sigma_{\mathrm{x}}}{8\pi\sigma_{\mathrm{y}}}, \tag{17}$$

$$\int p^2 dxdy = \frac{1}{4\pi\cdot\sigma_{\mathrm{x}}\cdot\sigma_{\mathrm{y}}}. \tag{18}$$

The identities above also hold when μ_{x} is replaced by μ_{y} and σ_{x} by σ_{y} and vice versa.

We derive

$$\sum_i \left(\frac{\partial p_i}{\partial \mu_{\mathrm{x}}}\right)^2 = \sum_i \int_{\text{pixel i}} a^2 p^2 \frac{(x-\mu_{\mathrm{x}})^2}{\sigma^4_{\mathrm{x}}} dxdy = \frac{a^2}{\sigma^4_{\mathrm{x}}}\int p^2(x-\mu_{\mathrm{x}})^2 dxdy = \frac{a^2}{\sigma^3_{\mathrm{x}}\sigma_{\mathrm{y}}}\frac{1}{8\pi}, \tag{19}$$

$$\sum_i p_i \left(\frac{\partial p_i}{\partial \mu_{\mathrm{x}}}\right)^2 = \frac{a^4}{\sigma^4_{\mathrm{x}}\sigma^2_{\mathrm{y}}}\frac{1}{36\pi 2}, \tag{20}$$

$$\sum_i \left(\frac{\partial p_i}{\partial \sigma^2_{\mathrm{x}}}\right)^2 = a^2\left(\frac{3}{64\pi\sigma_{\mathrm{y}}\sigma^5_{\mathrm{x}}} + \frac{1}{64\pi^3\sigma^3_{\mathrm{y}}\sigma^7_{\mathrm{x}}} - \frac{1}{32\pi^2\sigma^2_{\mathrm{y}}\sigma^6_{\mathrm{x}}}\right), \tag{21}$$

$$\sum_i p_i \left(\frac{\partial p_i}{\partial \sigma^2_{\mathrm{x}}}\right)^2 = a^4\left(\frac{1}{144\pi^2\sigma^2_{\mathrm{y}}\sigma^6_{\mathrm{x}}} + \frac{1}{192\pi^4\sigma^4_{\mathrm{y}}\sigma^8_{\mathrm{x}}} - \frac{1}{144\pi^3\sigma^3_{\mathrm{y}}\sigma^7_{\mathrm{x}}}\right). \tag{22}$$

By substituting these results in Supplementary Equation 12, the theoretical variances of the GMEs are obtained as

$$\mathrm{var}(\hat{\mu}_{\mathrm{x}}) \equiv \sigma^{*2}_{\hat{\mu}_{\mathrm{x}}}(z, N_{\mathrm{photons}}) = \frac{\sigma^2_{\mathrm{x}}(z)}{N_{\mathrm{photons}}}\left(\frac{16}{9} + \frac{b^2\sigma_{\mathrm{x}}(z)\sigma_{\mathrm{y}}(z)\cdot 8\pi}{a^2 N_{\mathrm{photons}}}\right), \tag{23}$$

$$\mathrm{var}(\hat{\mu}_{\mathrm{y}}) \equiv \sigma^{*2}_{\hat{\mu}_{\mathrm{y}}}(z, N_{\mathrm{photons}}) = \frac{\sigma^2_{\mathrm{y}}(z)}{N_{\mathrm{photons}}}\left(\frac{16}{9} + \frac{b^2\sigma_{\mathrm{y}}(z)\sigma_{\mathrm{x}}(z)\cdot 8\pi}{a^2 N_{\mathrm{photons}}}\right), \tag{24}$$

$$\mathrm{var}(\hat{\sigma}^2_{\mathrm{x}}) \equiv \sigma^{*2}_{\hat{\sigma}^2_{\mathrm{x}}}(z, N_{\mathrm{photons}}) \approx \frac{\sigma^4_{\mathrm{x}}(z)}{N_{\mathrm{photons}}}\left(\frac{256}{81} + \frac{64}{3}\frac{\sigma_{\mathrm{x}}(z)\sigma_{\mathrm{y}}(z)b^2}{a^2 N_{\mathrm{photons}}}\right), \tag{25}$$

$$\mathrm{var}(\hat{\sigma}^2_{\mathrm{y}}) \equiv \sigma^{*2}_{\hat{\sigma}^2_{\mathrm{y}}}(z, N_{\mathrm{photons}}) \approx \frac{\sigma^4_{\mathrm{y}}(z)}{N_{\mathrm{photons}}}\left(\frac{256}{81} + \frac{64}{3}\frac{\sigma_{\mathrm{x}}(z)\sigma_{\mathrm{y}}(z)b^2}{a^2 N_{\mathrm{photons}}}\right), \tag{26}$$

where the error in the later two expression is of the order $\sigma_{\mathrm{x}}^{-1}\sigma_{\mathrm{y}}^{-1}$. Since the width equals two times the standard deviation, the theoretical variance is described by Supplementary Equation 7.

Lateral uncertainty

It is generally assumed that the position of an observed photon follows a two-dimensional Gaussian distribution with mean $(\mu_{\mathrm{x}}, \mu_{\mathrm{y}})$ and variance $(\sigma^2_{\mathrm{x}}(z), \sigma^2_{\mathrm{y}}(z))$, where z denotes the height of the emitter. Thus, $\sigma^2_{\mathrm{x}}(z)$ and $\sigma^2_{\mathrm{y}}(z)$ describe the uncertainty in the (lateral) position of one single detected photon. Therefore $\sigma_{\mathrm{x}}(z)$ and $\sigma_{\mathrm{y}}(z)$ represent the so-called *low resolutions*, that is, the variance of one photon signal.
$\sqrt{\sigma^{*2}_{\hat{\mu}_{\mathrm{x}}}}$ and $\sqrt{\sigma^{*2}_{\hat{\mu}_{\mathrm{y}}}}$, see Supplementary Equations 23 and 24, are referred to as the *super resolution* in the x- and y-direction.

Let us evaluate a dSTORM image of a single dye on a bead. Let $\boldsymbol{y}_1, \boldsymbol{y}_2, ..., \boldsymbol{y}_k$ be the k localizations observed and $n_1, n_2, ..., n_k$ the numbers of photons that each estimate is based on. Recall the assumptions, for $i \in \{1, 2, ..., k\}$, that

$$Y_{x,\mathrm{i}} \sim \mathcal{N}\left(\mu_{\mathrm{x}}, \sigma^{*2}_{\hat{\mu}_{\mathrm{x}}}(z, n_i)\right), \quad (27)$$

$$Y_{\mathrm{y},i} \sim \mathcal{N}\left(\mu_{\mathrm{y}}, \sigma^{*2}_{\hat{\mu}_{\mathrm{y}}}(z, n_i)\right). \quad (28)$$

To validate that the variation of the assumed normal distribution is appropriate, the theoretical results are compared to the observed data (dSTORM images). Since every localization is based on a different number of photon signals the localizations are not identically distributed.

By rescaling all the localizations by $\sqrt{n_i}$, k normal random variables with means $\sqrt{n_i}\mu_{\mathrm{x}}$ and variances that differ by a $\frac{1}{n_i^2}$ term are obtained. Next, the real x-coordinate of the dye is estimated as

$$\hat{\mu}_{\mathrm{x}} = \frac{1}{\sum_{i=1}^{k-1}\sqrt{n_i}} \sum_{i=1}^{k} \sqrt{n_i} \cdot y_{\mathrm{x},i}. \quad (29)$$

Since the observations are now independent and identically distributed with equal means, this estimate is consistent by Kolmogorov's strong law of large numbers, see e.g. [23, Theorem 2.3.10]. By subtracting this estimate from every realization,realizations from a asymptotic normal distribution with zero mean and variance $\sigma^{*2}_{\hat{\mu}_{\mathrm{x}}}(z, n_i)$ are obtained. Therefore,

$$(Y_{x,\mathrm{i}} - \hat{\mu}_{\mathrm{x}})^2 \xrightarrow{d} \sigma^{*2}_{\hat{\mu}_{\mathrm{x}}}(z, n_i)\chi^2_1, \quad (30)$$

where χ^2_1 is a Chi-squared distributed random variable with one degree of freedom.

With use of the $\hat{\mu}_{\mathrm{z}}$ estimated from the single molecule data the quadratic distances based on the Chi-square distribution are simulated and in turn compared to the observed data. By doing so, we compare several statistical measures of the data with the expected value based on the theoretical results. In Supplementary Table 4 the estimated first quantile (Q1), median, mean, third quantile (Q3) and variance for the observed squared differences and the simulated squared differences in both lateral directions are presented.

	Q1	Median	Mean	Q3	Variance
data, x	20.18	94.72	268.50	309.20	207807.43
theory, x	13.30	65.11	195.40	223.50	121015.66
data, y	18.97	95.58	280.20	311.60	236373.84
theory, y	13.58	66.12	204.60	230.70	137461.80

Supplementary Table 4: Squared differences of lateral coordinates

We conclude that the theoretical estimation errors *underestimate* the variability found in the data. It is well known that drift plays a role in the imaging [10]. Whether due to environmental fluctuations, a suboptimality in the microscope setup or other experimental factors, drift on the nanometer scale is notoriously difficult to prevent. Therefore, super-resolution measurements need to be corrected for this phenomenon. Importantly, the drift correction is done after a two-dimensional Gaussian distribution is fitted to the photon cloud. Since the drift within an active period is negligible, only the localization is corrected for drift and not every single photon signal. This correction consists of subtracting an estimate for the drift, at the moment t, at which the signals the localizations is based on are measured, of the estimated position. This estimate is referred to as $B(t)$. The misfit to the data is partly explained by this drift. Therefore, a new model is defined as

$$Y_{x,\mathrm{i}}(t) \sim \mathcal{N}\left(\mu_{\mathrm{x}}, \sigma^{*2}_{\hat{\mu}_{\mathrm{x}}}(z, n_i)\right) + B_{\mathrm{x}}(t), \tag{31}$$

$$Y_{\mathrm{y},i}(t) \sim \mathcal{N}\left(\mu_{\mathrm{y}}, \sigma^{*2}_{\hat{\mu}_{\mathrm{y}}}(z, n_i)\right) + B_{\mathrm{y}}(t). \tag{32}$$

The estimate of $B(t)$ is based on a collection of bright particles that emit photons continuously (fiducial marker). This estimate is modelled with a normal distribution $\mathcal{N}(B(t), \sigma^2_{\mathrm{drift}})$, where $\sigma^2_{\mathrm{drift}}$ describes the error in measuring the position of the bright particle. Experimentally, every image contains several (ideally 5-10) fiducial markers. In every frame, a location estimate for each of these $B_i(t)$ is obtained using a normal GME, and $B_i(0)$ is subtracted to yield an estimated displacement of marker i. The average displacement of all markers in an image ($N^{-1}\sum_{i=1}^{N} B_i(t) - B_i(0)$, for $t \geq 0$) is the drift correction for that image. The standard error of this drift correction is estimated as

$$\sqrt{\frac{N-1}{N}\sum_{i=1}^{N}\left(B_i(t) - B_i(0) - \left(N^{-1}\sum_{i=1}^{N} B_i(t) - B_i(0)\right)\right)^2} \tag{33}$$

and provides a measure of the error in the fiducial marker localization, and hence the drift correction. This measure corresponds to $\sigma^2_{\mathrm{drift}}$. As a result the model becomes

$$Y_{x,\mathrm{i}}(t) \sim \mathcal{N}\left(\mu_{\mathrm{x}}, \sigma^{*2}_{\hat{\mu}_{\mathrm{x}}}(z, n_i) + \sigma^2_{\mathrm{drift}}\right), \tag{34}$$

$$Y_{\mathrm{y},i}(t) \sim \mathcal{N}\left(\mu_{\mathrm{y}}, \sigma^{*2}_{\hat{\mu}_{\mathrm{y}}}(z, n_i) + \sigma^2_{\mathrm{drift}}\right). \tag{35}$$

The additional experimental variance term due to drift correction $\sigma^2_{\mathrm{drift}}$, as estimated from the variability of the fiducial marker localizations, amounts to $\hat{\sigma}^2_{\mathrm{drift}} = 5.5^2$. The validation procedure is repeated for this corrected model. The summary statistics of the new simulated data are presented in Supplementary Table 5.

	Q1	Median	Mean	Q3	Variance
data, x	20.18	94.72	268.50	309.20	207807.43
theory+drift, x	17.88	81.09	224.30	266.30	147744.78
data, y	18.97	95.58	280.20	311.60	236373.84
theory+drift, y	17.35	83.00	235.20	270.60	176373.95

Supplementary Table 5: Squared differences of lateral coordinates

We conclude that the combination of the derived estimation error with the drift is not enough to explain all the variability observed in the data. Before this model is further expanded in subsection the axial uncertainty is discussed in the next subsection.

Axial uncertainty

In subsection the methods used to estimate the z-coordinate of a dye were explained. The uncertainty in this estimate is a consequence of the uncertainty in estimating the x- and y-widths. Via the projection in the square root space, see subsection , this uncertainty propagates.

Uncertainty in width estimates

Let $\boldsymbol{w}_1, \boldsymbol{w}_2, ..., \boldsymbol{w}_k$ be the k observed widths, where $\boldsymbol{w}_i = (w_{\mathrm{x},i}, w_{\mathrm{y},i})$ for $i \in \{1, 2, ..., k\}$. Here we assume

$$(W_{\mathrm{x},i}/2)^2 \sim \mathcal{N}\left(\sigma_{\mathrm{x}}^2(z), \sigma_{\hat{\sigma}_{\mathrm{x}}^2}^{*2}(z, n_i)\right), \tag{36}$$

$$(W_{\mathrm{y},i}/2)^2 \sim \mathcal{N}\left(\sigma_{\mathrm{y}}^2(z), \sigma_{\hat{\sigma}_{\mathrm{y}}^2}^{*2}(z, n_i)\right), \tag{37}$$

based on the expression of the GME and central limit theorem arguments. The factors 1/2 appear since the width equals twice the standard deviation. The propagation of errors are described using Taylor expansions by application of the Delta-method, see e.g. [25, Chapter 2], which results in

$$W_{\mathrm{x},i} \sim \mathcal{N}\left(2\sigma_{\mathrm{x}}(z), \frac{\sigma_{\hat{\sigma}_{\mathrm{x}}^2}^{*2}(z, n_i)}{\sigma_{\mathrm{x}}^2(z)}\right), \tag{38}$$

$$W_{\mathrm{y},i} \sim \mathcal{N}\left(2\sigma_{\mathrm{y}}(z), \frac{\sigma_{\hat{\sigma}_{\mathrm{y}}^2}^{*2}(z, n_i)}{\sigma_{\mathrm{y}}^2(z)}\right). \tag{39}$$

We validate the theoretical variance expression using the squared difference with respect to the widths on the calibration curve. $z = \mu_{\mathrm{z}}$ is estimated as

$$\hat{\mu}_{\mathrm{z}} = \frac{1}{\sum_{i=1}^{k-1} \sqrt{n_i}} \sum_{i=1}^{k} \sqrt{n_i} \cdot y_{\mathrm{z},i}. \tag{40}$$

Note that by the continuous mapping theorem, see e.g. [25, Chapter 2], $2\sigma_{\mathrm{x}}(\hat{z}) \xrightarrow{d} 2\sigma_{\mathrm{x}}(z)$. Therefore,

$$(W_{\mathrm{x},i} - 2\sigma_{\mathrm{x}}(\hat{z}))^2 \xrightarrow{d} \frac{\sigma_{\hat{\sigma}_{\mathrm{x}}^2}^{*2}(z, n_i)}{\sigma_{\mathrm{x}}^2(z)} \chi_1^2. \tag{41}$$

In Supplementary Table 6 the estimated statistical measures for the observed and simulated squared differences of the width with the actual value on the calibration curve are shown. We again conclude that the theoretical expressions do not match the variability found in the data. Note that estimates of the width are not influenced by the drift since these do not depend on the exact x- and y-position.

The expression for the axial coordinate estimate $\hat{z}(w_{\mathrm{x}}(z), w_{\mathrm{y}}(z))$ based on the relation between z and w_{x} and w_{y}, respectively, presented as Supplementary Equations 7 and 8, is difficult to handle; the projection is performed in the square root space and the projection is performed on a parametric equation. Therefore, an analytical expression for this relation is out of reach. However, given a specific coordinate $w_{\mathrm{x}}', w_{\mathrm{y}}'$, it is simple to compute the corresponding estimate for z. Therefore, we proceed by adding noise (steps of 0.1) to the

	1st Qu.	Median	Mean	3rd Qu.	variance
data width, x	123.70	556.50	1020.00	1500.00	1469533.38
theory width, x	30.46	145.50	454.70	503.10	718162.39
data width, y	99.63	452.60	897.80	1290.00	1281538.85
theory width, y	28.05	145.20	484.30	524.00	863028.66

Supplementary Table 6: Squared differences of the widths with calibration curve at $\hat{z}$

observed width belonging to z from -165 to 165 (steps of 0.5), and compute the $\hat{z}$. This way the function values $\hat{z}(w_\mathrm{x}(z), w_\mathrm{y}(z))$ for a wide range of possible width combinations are sampled. Next, $\hat{z}(w_\mathrm{x}(z), w_\mathrm{y}(z))$ is approximated with

$$z + w_\mathrm{x}(z) \cdot (\alpha_\mathrm{x} + z \cdot \beta_\mathrm{x}) + w_\mathrm{y}(z) \cdot (\alpha_\mathrm{y} + z \cdot \beta_\mathrm{y}). \quad (42)$$

The method of least squares is used to find $\alpha_\mathrm{x}, \alpha_\mathrm{y}, \beta_\mathrm{x}, \beta_\mathrm{y}$ that gives rise to the most appropriate approximation. The estimated covariates are

$$\hat{z} \approx z + (\hat{w}_\mathrm{x}(z) - w_\mathrm{x}(z)) \cdot (1.201 - z \cdot 0.001) + (\hat{w}_\mathrm{y}(z) - w_\mathrm{y}(z)) \cdot (-1.219 - z \cdot 0.013). \quad (43)$$

Since $\hat{w}_\mathrm{x}(z) - w_\mathrm{x}(z)$ and $\hat{w}_\mathrm{y}(z) - w_\mathrm{y}(z)$ are uncorrelated we conclude that

$$\mathrm{var}(\hat{\mu}_\mathrm{z}) = \mathrm{var}(\hat{w}_\mathrm{x}(z)) \cdot (1.201 - z \cdot 0.001)^2 + \mathrm{var}(\hat{w}_\mathrm{y}(z)) \cdot (-1.219 - z \cdot 0.013)^2. \quad (44)$$

The variance of $\hat{w}_\mathrm{x}$ and $\hat{y}_\mathrm{x}$ have already been discussed in detail, see Supplementary Equation 38. Here the drift should be taken into account as well, since the different z estimates are collected over time. Therefore,

$$y_{\mathrm{z},i}(t) \sim \mathcal{N}\left(\mu_\mathrm{z}, \mathrm{var}(\hat{\mu}_\mathrm{z}) + \sigma^2_{\mathrm{drift,z}}\right). \quad (45)$$

Based on the displacement of the fiducial marker, $\hat{\sigma}^2_{\mathrm{drift,z}} = 19.5^2$. In Supplementary Table 7 the observed squared difference are compared to the simulated squared difference. We

	1st Qu.	Median	Mean	3rd Qu.	variance
data	182.20	843.60	1919.00	2507.00	8134350.10
theory	83.47	416.20	1295.00	1481.00	5381073.60
theory+drift	136.90	639.60	1657.00	1977.00	7368992.19

Supplementary Table 7: Squared differences of the z-location with the estimated real z coordinate.

conclude that the drift partly explains the variation present in (the axial coordinates of) the data. However, there seems to be a part of the variance that is not explained by the drift. It is remarkable that this underestimation of the variance is smaller than for the variance of the widths, see Supplementary Table 6.

Mixed Normal distribution

In subsection , it was shown how the assumed normal distribution underestimates the variance of the estimates in the lateral direction and the variance of the estimates of the widths. As a result the variance for the axial coordinate estimate is also underestimated. Here the values derived in subsection will be used.

A new model based on a mixed normal distribution is proposed, such that

$$Y_{\mathrm{x},j,i} \sim \mathcal{N}(\mu_{x,j} + X_{\mu_\mathrm{x},j}, \sigma_\mathrm{x}^2(z_j) + X_{\sigma_\mathrm{x}^2,j}), \text{ for } i = 1, 2, ..., N_{\mathrm{photons},j}, \tag{46}$$

with $X_{\mu_\mathrm{x},j}$ and $X_{\sigma_\mathrm{x}^2,j}$ zero-mean random variables with variances $\Sigma_{1,x}$ and $\Sigma_{2,\mathrm{x}}$. Note that the distribution of the X is not of interest here. The distributions of the localizations originating from the same dye may differ e.g., as a result of changing dipole orientations or coupling of the fluorophore excitation dipole to nearby interfaces [19]. By the law of total variation, see e.g. [27, Exercise 4.1.7], for two random variables X and Y such that $\mathrm{var}(Y) < \infty$,

$$\mathrm{var}(Y) = \mathbb{E}\left[\mathrm{var}\left(Y|X\right)\right] + \mathrm{var}\left(\mathbb{E}\left[Y|X\right]\right). \tag{47}$$

The law of total variation is applied to derive the variance of the location estimate of emitter j. That $\mathrm{var}(\hat{\mu}_\mathrm{x})$, which depends on (z, N_photons) can be approximated as

$$\begin{aligned}
\mathrm{var}(\hat{\mu}_\mathrm{x}) =& \mathbb{E}\left[\mathrm{var}(\hat{\mu}_\mathrm{x}|X_{\mu_\mathrm{x}}, X_{\sigma_\mathrm{x}^2})\right] + \mathrm{var}\left(\mathbb{E}\left[\hat{\mu}_\mathrm{x}|X_{\mu_\mathrm{x}}, X_{\sigma_\mathrm{x}^2}\right]\right),\\
=& \mathbb{E}\left[\frac{\sigma_\mathrm{x}^2(z) + \Sigma_{2,\mathrm{x}}}{N_\mathrm{photons}}\left(\frac{16}{9} + \frac{b^2\sqrt{(\sigma_\mathrm{x}^2(z) + \Sigma_{2,\mathrm{x}})(\sigma_\mathrm{y}^2(z) + \Sigma_{2,\mathrm{x}})\cdot 8\pi}}{a^2 N_\mathrm{photons}}\right)\right]\\
&+ \mathrm{var}\left(\mathbb{E}\left[\hat{\mu}_\mathrm{x}|X_{\mu_\mathrm{x}}\right]\right),\\
\approx& \mathbb{E}\left[\frac{\sigma_\mathrm{x}^2(z) + \Sigma_{2,\mathrm{x}}}{N_\mathrm{photons}}\left(\frac{16}{9} + \frac{b^2\sigma_\mathrm{x}(z)\sigma_\mathrm{y}(z)\cdot 8\pi}{a^2 N_\mathrm{photons}}\right)\right] + \mathrm{var}\left(\mathbb{E}\left[\hat{\mu}_\mathrm{x}|X_{\mu_\mathrm{x}}\right]\right),\\
=& \sigma_{\hat{\mu}_\mathrm{x}}^{*2}(z, N_\mathrm{photons}) + \mathrm{var}(\mu_\mathrm{x} + X_{\mu_\mathrm{x}}),\\
=& \sigma_{\hat{\mu}_\mathrm{x}}^{*2}(z, N_\mathrm{photons}) + \Sigma_{1,x}.
\end{aligned} \tag{48}$$

Since $\Sigma_{2,\mathrm{x}}$ will be small compared to $\sigma_\mathrm{x}^2(z)$ an approximation is used. Similarly, the variance of the estimate for $\sigma_\mathrm{x}^2(z_j)$ is derived as

$$\begin{aligned}
&\mathrm{var}(\hat{\sigma}_\mathrm{x}^2) = \mathbb{E}\left[\mathrm{var}(\hat{\sigma}_\mathrm{x}^2(z)|X_{\mu_\mathrm{x}}, X_{\sigma_\mathrm{x}^2})\right] + \mathrm{var}\left(\mathbb{E}\left[\hat{\sigma}_\mathrm{x}^2(z)|X_{\mu_\mathrm{x}}, X_{\sigma_\mathrm{x}^2}\right]\right)\\
&= \mathbb{E}\left[\frac{(\sigma_\mathrm{y}^2(z) + \Sigma_{2,\mathrm{x}})^2}{N_\mathrm{photons}}\left(\frac{256}{81} + \frac{64}{3}\frac{\sqrt{(\sigma_\mathrm{x}^2(z) + \Sigma_{2,\mathrm{x}})(\sigma_\mathrm{y}^2(z) + \Sigma_{2,\mathrm{x}})}b^2}{a^2 N_\mathrm{photons}})\right)\right] + \mathrm{var}(\sigma_\mathrm{x}^2 + \Sigma_{2,\mathrm{x}})\\
&\approx \mathbb{E}\left[\frac{(\sigma_\mathrm{x}^4(z) + 2\sigma_\mathrm{x}^2\cdot\Sigma_{2,\mathrm{x}} + \Sigma_{2,\mathrm{x}}^2)}{N_\mathrm{photons}}\left(\frac{256}{81} + \frac{64}{3}\frac{\sigma_\mathrm{x}(z)\sigma_\mathrm{y}(z)b^2}{a^2 N_\mathrm{photons}}\right)\right] + \mathrm{var}(\sigma_\mathrm{x}^2 + \Sigma_{2,\mathrm{x}})\\
&= \mathbb{E}\left[\frac{(\sigma_\mathrm{x}^4(z) + 2\sigma_\mathrm{x}^2\cdot\Sigma_{2,\mathrm{x}} + \Sigma_{2,\mathrm{x}}^2)}{N_\mathrm{photons}}\left(\frac{256}{81} + \frac{64}{3}\frac{\sigma_\mathrm{x}(z)\sigma_\mathrm{y}(z)b^2}{a^2 N_\mathrm{photons}}\right)\right] + \mathrm{var}(\Sigma_{2,\mathrm{x}})\\
&\\
&= \sigma_{\hat{\sigma}_\mathrm{x}^2}^{*2}(z, N_\mathrm{photons}) + \left(1 + \frac{256}{81 N_\mathrm{photons}} + \frac{64}{3}\frac{\sigma_\mathrm{x}(z)\sigma_\mathrm{y}(z)b^2}{a^2 N_\mathrm{photons}^2}\right)\mathrm{var}(\Sigma_{2,\mathrm{x}})\\
&\approx \sigma_{\hat{\sigma}_\mathrm{x}^2}^{*2}(z, N_\mathrm{photons}) + \mathrm{var}(\Sigma_{2,\mathrm{x}}).
\end{aligned} \tag{49}$$

To estimate $\Sigma_{1,x}$ and $\Sigma_{1,y}$, the sum of squares of the differences between the estimated squared difference and $\text{var}(\hat{\mu}_\text{x})\,(\hat{z}, N_\text{photons})$ is minimized. For $\Sigma_{2,\text{x}}$ and $\Sigma_{2,\text{y}}$, respectively, to obtain a robust estimate, the sum of absolute differences between the estimated squared difference and $\text{var}(\hat{\sigma}^2_\text{x})\,(\hat{z}, N_\text{photons})$ is minimized. The global estimates of these quantities are given in Supplementary Table 8 below.

Parameter	Estimate
$\Sigma_{1,x}$	7.82^2
$\Sigma_{1,y}$	8.20^2
$\Sigma_{2,\text{x}}$	17.46^2
$\Sigma_{2,\text{y}}$	15.21^2

Supplementary Table 8: Mixture parameter estimates.

Based on these estimates the observed squared difference are compared to the simulated ones based on the new variance expressions. The statistics are given in Supplementary Table 9.

	1st Qu.	Median	Mean	3rd Qu.	variance
data, x	20.18	94.72	268.50	309.20	207807.43
mixture+drift,x	23.07	105.30	265.10	318.90	185802.98
data, y	18.97	95.58	280.20	311.60	236373.84
mixture+drift,y	23.24	109.60	280.80	334.10	217346.83
data width,x	123.70	556.50	1020.00	1500.00	1469533.38
mixture,wx	69.54	315.70	755.90	933.20	1443789.45
data width, y	99.63	452.60	897.80	1290.00	1281538.85
mixture,wy	59.52	275.00	712.90	860.00	1453860.22
data,z	182.20	843.60	1919.00	2507.00	8134350.10
mixture+drift,z	240.90	1084.00	2472.00	3083.00	14132578.52

Supplementary Table 9: Squared differences; comparison data and simulations.

By comparing Supplementary Table 9 to Supplementary Tables 5 and 6, we conclude that the variability in the lateral dimension is described satisfactorily, while the variability in the widths is underestimated. However, this model introduces more variability in the axial dimension than is observed in the data. The median of the axial absolute differences equal 25.3nm for the model with drift, 29.0nm for the data and 32.9nm for the mixture model. The observed absolute differences and the expected values based on the different theoretical models are presented in Supplementary Figures 7, 8 and 9. Despite the small residual inaccuracies, the model presented in Supplementary Equation 46 succeeds in capturing the experimentally observed measurement uncertainties in great detail. The analysis using the mixture model will be conservative since the estimated variability used in the axial dimension will be slightly higher than in reality.

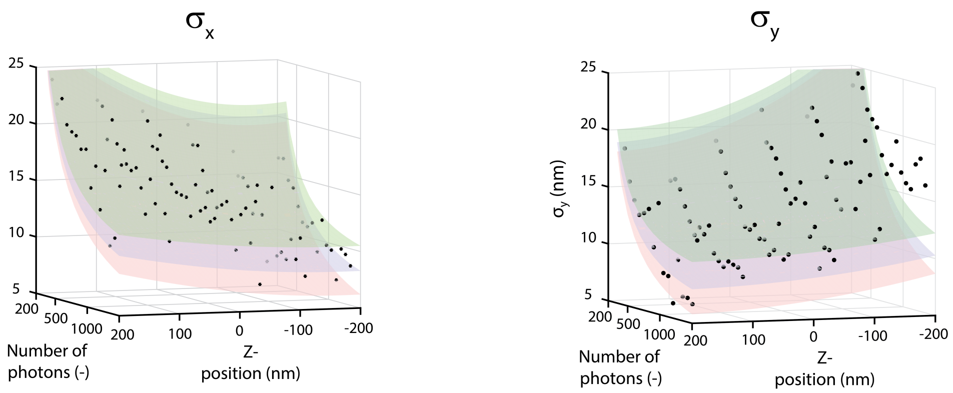

Supplementary Figure 7: Observed absolute differences between localization locations and the estimated source location averaged over 75nm × 75nm photons bins (black). The expected values based on the theoretical- (red), drift extension- (blue) and mixed normal- (green) models for the lateral standard deviation.

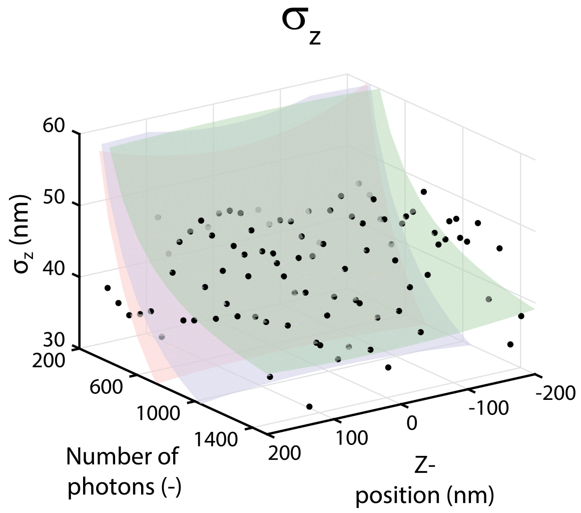

Supplementary Figure 8: Observed absolute differences between localization locations and the estimated source location averaged over 75nm × 75nm photons bins (black). The expected values based on the theoretical- (red), drift extension- (blue) and mixed normal- (green) models for the axial standard deviation.

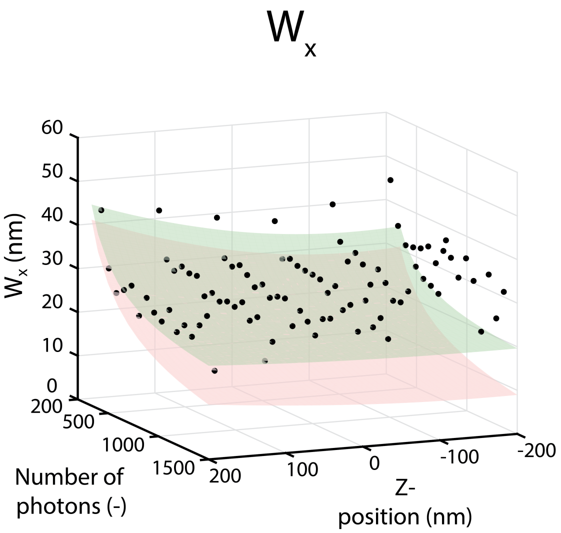

Supplementary Figure 9: Observed absolute differences between localization widths and the widths based on the estimated z-coordinate and observed number of photons averaged over 75nm $\times$ 75nm photons bins (black). The expected values based on the theoretical- (red) and mixed normal- (green) models for the standard deviation of the widths in both lateral dimensions.

Estimators for the number of dyes

In this subsection two possible estimators for the number of dyes on a bead, given the number of observed localizations Q, are compared. Based on 1000 simulated beads the mean squared error (MSE), the average of the squared estimation error, for the three different bead densities, for both methods is computed.

Division by average. An intuitive and simple estimate for the number of dyes is

$$\mathrm{DBA}(Q) = \frac{Q}{\mathbb{E}(B)} = Q \cdot \hat{p}_{\text{bleach}}. \tag{50}$$

Compound negative binomial distribution. The second method is based on the fit of the negative binomial distribution for the number of localizations data. This allows us to obtain a probability distribution for the number of emitters (at equilibrium time) on a bead, given the observed blinks. The probability generating function of B is given by $G_B(z) = \frac{zp}{1-z(1-p)}$ and $G_{N_{\text{dark}}}(z) = \frac{p}{1-z(1-p)}$, where $p = p_{\text{bleach}}$. The probability generating function of X, where $X \sim \mathrm{NB}(p, r)$ is given by $G_{\mathrm{x}}(z) = \left(\frac{1-p}{1-zp}\right)^r$. In other words a sum of k independent N_{dark} random variables is distributed as negative binomial distributed, $\sum_{i=0}^{k} N_{\text{dark}} \sim \mathrm{NB}(1 - p_{\text{bleach}}, k)$. Recall also that $B = N_{\text{dark}} + 1$. These facts give us

$$\begin{aligned}
\mathbb{P}\left(X_j = k \;\middle|\; \sum_{i=1}^{X_j} B_i = q_j\right) &= \mathbb{P}\left(X_j = k \;\middle|\; \sum_{i=1}^{X_j} N_{\text{dark},i} = q_j - X_j\right) \\
&= \frac{\mathbb{P}\left(X = k, \sum_{i=1}^{X} N_{\text{dark},i} = q_j - X\right)}{\mathbb{P}\left(\sum_{i=1}^{X} N_{\text{dark},i} = q_j - X\right)} \\
&= \frac{\mathbb{P}(X = k) \cdot \mathbb{P}\left(\sum_{i=1}^{k} N_{\text{dark},i} = q_j - k\right)}{\mathbb{P}\left(\sum_{i=1}^{X} N_{\text{dark},i} = q_j - X\right)} \\
&= \frac{\mathbb{P}(X = k) \cdot \mathbb{P}\left(\sum_{i=1}^{k} Q_i = q_j - k\right)}{\sum_{l=0}^{q_j} \mathbb{P}\left(\sum_{m=1}^{l} B_m = q_j - l\right) \cdot \mathbb{P}(X = l)}.
\end{aligned} \tag{51}$$

Using this expression, the number of dyes is estimated. $CNB(Q)$ equals the MLE estimate for X given Q, i.e.

$$\mathrm{CNB}(Q) = \underset{1 \le k \le Q}{\operatorname{argmax}}\, \mathbb{P}\left(X = k \;\middle|\; \sum_{i=1}^{X} B_i = Q\right). \tag{52}$$

Results. The square roots of the computed MSE and the expected number of dyes based on the $NB(r, p)$ distribution, see Supplementary Table 2, are presented in Supplementary Table 10. We conclude that the estimator based on the compound negative binomial distribution is in expectation more precise than the other estimator, but the difference is very small. Still we recommend to use the estimator based on compound negative binomial distributions.

Bead density	DBA	CNB	$\mathbb{E}[X]$
L	2.62	2.41	9.94
M	5.76	5.59	52.84
H	12.70	12.48	215.34

Supplementary Table 10: Square root of MSE estimates

Estimate for the real position of the dyes

In subsection an estimator for the number of emitters for a specific image has been determined. Next, the assignment of the localizations to these emitters is described. Since the positions of the emitters are not known, these need to be estimated at the same time. This analysis is often referred to as 'clustering', see for example [14, Chapter 10] and [15, Paragraph 14.3].

Mathematical theory

For a detailed treatment of the material of this subsection, we refer the reader to [14]. We will assume that the following holds:

(A1) The localizations originate from a known number N of emitters.

(A2) The localization labels H_j are unknown.

(A3) The positions for the N emitters (parameter vectors), $\boldsymbol{\theta} = (\boldsymbol{\mu}_1, ..., \boldsymbol{\mu}_N)$, are unknown.

(A4) The prior probabilities, $\mathbb{P}(H_j = i)$ for each emitter i are known.

(A5) The form for the class-conditional probability densities $f_{\mathbf{Y}|H_j,\boldsymbol{\theta}}(\mathbf{y}_j)$ is known.

In (A2) $H_j = i$ means that localization $j$originates from emitter i. Let us focus on the prior probability of localization $j$coming from emitter i, $j = 1, ..., n$, $i = 1, ..., N$. Emitters are identical and independent from one another. Since the positions of the different emitters are unknown, every emitter is equally probable to be the source of a certain localization. Therefore, the prior probability is uniform over all emitters, that is $\mathbb{P}(H_j = i) = 1/N$ for all i.

The probability density function of the position of a localization given the positions of the emitters is

$$f_{\mathbf{Y}_j|\boldsymbol{\theta}}(\mathbf{y}_j) = \sum_{k=1}^{N} f_{\mathbf{Y}_j|H_j(\mathbf{y}_j)=k,\boldsymbol{\theta}}(\mathbf{y}_j) \cdot \mathbb{P}(H_j = k). \tag{53}$$

The likelihood of the trivariate (x-,y- and z- coordinate) normal distribution given the n localizations is

$$\begin{aligned} L(\boldsymbol{\theta}|\mathbf{y_1}, \mathbf{y_2}, ..., \mathbf{y_n}) &= \prod_{j=1}^{n} f_{\mathbf{Y}_j|\boldsymbol{\theta}}(\mathbf{y}_j) \\ &= \prod_{j=1}^{n} \sum_{k=1}^{N} f_{\mathbf{Y}_j|H_j(\mathbf{y}_j)=k,\boldsymbol{\mu}_k}(\mathbf{y}_j) \cdot \mathbb{P}(H_j = k). \end{aligned} \tag{54}$$

The maximum-likelihood estimate $\hat{\boldsymbol{\theta}}$ is the maximizer of Supplementary Equation 54. It is convenient to define the log-likelihood as

$$l(\boldsymbol{\theta}|\mathbf{y}_1, \mathbf{y}_2, ..., \mathbf{y}_\text{n}) = \ln L(\boldsymbol{\theta}|\mathbf{y}_1, \mathbf{y}_2, ..., \mathbf{y}_\text{n}) \tag{55}$$

$$= \sum_{j=1}^{n} \ln f_{\mathbf{Y}_j|\boldsymbol{\theta}}(\mathbf{Y}_j), \tag{56}$$

so that $\hat{\boldsymbol{\theta}}$ maximizes Supplementary Equation 55. The derivatives with respect to each component of $\boldsymbol{\theta}$ equal zero for the estimate of $\hat{\boldsymbol{\theta}}$ that maximizes Supplementary Equation 55. The derivative equals

$$\begin{aligned}\frac{\partial}{\partial \boldsymbol{\mu}_i} l(\boldsymbol{\theta}|\mathbf{y}_1, \mathbf{y}_2, ..., \mathbf{y}_\mathrm{n}) &= \sum_{j=1}^{n} \frac{1}{f_{\mathbf{Y}_j|\boldsymbol{\theta}}(\mathbf{y}_j)} \cdot \frac{d}{d\boldsymbol{\mu}_i} f_{\mathbf{Y}_j|\boldsymbol{\theta}}(\mathbf{y}_j) \\ &= \sum_{j=1}^{n} \frac{1}{f_{\mathbf{Y}_j|\boldsymbol{\theta}}(\mathbf{y}_j)} \cdot \frac{d}{d\boldsymbol{\mu}_i} \sum_{k=1}^{N} f_{\mathbf{Y}_j|H_j=k,\boldsymbol{\mu}_i}(\mathbf{y}_j) \cdot \mathbb{P}(H_j = k). \end{aligned} \tag{57}$$

The probability for a certain localization to come from a specific emitter given the location of this emitter is computed as

$$\begin{aligned}\mathbb{P}(H_j = i|\boldsymbol{\theta}) &= \frac{f_{\mathbf{Y}_j|H_j,\boldsymbol{\theta}(\mathbf{y}_j)} \cdot \mathbb{P}(H_j = i)}{\sum_{i=1}^{N} f_{\mathbf{Y}_j|H_j,\boldsymbol{\theta}}(\mathbf{y}_j) \cdot \mathbb{P}(H_j = i)} \\ &= \frac{f_{\mathbf{Y}_j|H_j,\boldsymbol{\theta}(\mathbf{y}_j)} \cdot \mathbb{P}(H_j = i)}{f_{\mathbf{Y}_j|\boldsymbol{\theta}}(\mathbf{y}_j)}. \end{aligned} \tag{58}$$

The derivative with respect to $\boldsymbol{\mu}_i$ is evaluated in Supplementary Equation 57. The densities that are not functions of $\boldsymbol{\mu}_i$ do not contribute so that,

$$\begin{aligned}\frac{d}{d\boldsymbol{\mu}_i} l(\boldsymbol{\theta}|\mathbf{y_1}, \mathbf{y_2}, ..., \mathbf{y_n},) &= \sum_{j=1}^{n} \frac{1}{f_{\mathbf{Y}_j|\boldsymbol{\theta}}(\mathbf{y}_j)} \cdot \frac{d}{d\boldsymbol{\mu}_i} f_{\mathbf{Y}_j|H_j,\boldsymbol{\mu}_i}(\mathbf{y}_j) \cdot \mathbb{P}(H_j = i) \\ &= \sum_{j=1}^{n} \frac{\mathbb{P}(H_j = i)}{f_{\mathbf{Y}_j|\boldsymbol{\theta}}(\mathbf{y}_j)} \cdot \frac{d}{d\boldsymbol{\mu}_i} f_{\mathbf{Y}_j|H_j,\boldsymbol{\mu}_i}(\mathbf{y}_j). \end{aligned} \tag{59}$$

Now Supplementary Equation 58 gives

$$\begin{aligned}\frac{d}{d\boldsymbol{\mu}_i} l(\boldsymbol{\theta}|\mathbf{y}_1, \mathbf{y}_2, ..., \mathbf{y}_\mathrm{n}) &= \sum_{j=1}^{n} \frac{\mathbb{P}(H_j = i|\boldsymbol{\theta})}{f_{\mathbf{Y}_j|H_j,\boldsymbol{\theta}}(\mathbf{y}_j)} \cdot \frac{d}{d\boldsymbol{\mu}_i} f_{\mathbf{Y}_j|H_j,\boldsymbol{\mu}_i}(\mathbf{y}_j) \\ &= \sum_{j=1}^{n} \mathbb{P}(H_j = i|\boldsymbol{\theta}) \frac{d}{d\boldsymbol{\mu}_i} \ln f_{\mathbf{Y}_j|H_j,\boldsymbol{\mu}_i}(\mathbf{y}_j). \end{aligned} \tag{60}$$

In other words the maximum-likelihood estimate for $\boldsymbol{\mu}_i$ must satisfy

$$\sum_{j=1}^{n} \mathbb{P}(H_j = i|\boldsymbol{\theta}) \frac{d}{d\boldsymbol{\mu}_i} \ln f_{\mathbf{Y}_j|H_j,\boldsymbol{\mu}_i}(\mathbf{y}_j) = 0. \tag{61}$$

The position of a localization, $\boldsymbol{\theta} = (\boldsymbol{\mu}_1, \boldsymbol{\mu}_2, ..., \boldsymbol{\mu}_N)$, where $\boldsymbol{\mu}_i \in \mathbb{R}^3$, given the position of the emitter, follows a trivariate normal distribution. Let $\Sigma_j = I \cdot \boldsymbol{\sigma}_j$ as introduced in subsection . Now the class-conditional probability density, likelihood of observations given labels localizations and position emitters, $f_{\mathbf{Y}_j|H_j,\boldsymbol{\theta}}(\mathbf{y}_j)$ equals

$$f_{\mathbf{Y}_j|H_j,\boldsymbol{\theta}}(\mathbf{Y}_j) = (2\pi)^{-\frac{3}{2}} |\Sigma_j|^{-\frac{1}{2}} \exp\left(-\frac{1}{2}(\mathbf{Y}_j - \boldsymbol{\mu}_{H_j})' {\Sigma_j}^{-1} (\mathbf{Y}_j - \boldsymbol{\mu}_{H_j})\right). \tag{62}$$

Therefore,

$$\ln f_{\mathbf{Y}_j|H_j=i,\boldsymbol{\theta}}(\mathbf{y}_j) = -\frac{3}{2}\ln 2\pi - \frac{1}{2}\ln|\Sigma_j| - \frac{1}{2}\cdot(\mathbf{Y}_j - \boldsymbol{\mu}_i)'{\Sigma_j}^{-1}(\mathbf{Y}_j - \boldsymbol{\mu}_i), \tag{63}$$

and

$$\frac{d}{d\boldsymbol{\mu}_i}\ln f_{\mathbf{Y}_j|H_j=i,\boldsymbol{\theta}}(\mathbf{y}_j) = {\Sigma_j}^{-1}(\mathbf{Y}_j - \boldsymbol{\mu}_i). \tag{64}$$

In reality all the emitters should lay on the sphere, in other words $(\boldsymbol{\mu}_i - \mathbf{m})^T \cdot (\boldsymbol{\mu}_i - \mathbf{m}) = r^2$ where $\mathbf{m}$ equals the midpoint of the bead of interest and r equals the radius. Let $\mathbf{m} = (0,0,0)$. A Lagrange multiplier λ_i is introduced in Supplementary Equation 55. This gives N extra constraints of the form $\boldsymbol{\mu}_i^T\boldsymbol{\mu}_i - r^2 = 0$. As a consequence the likelihood equals

$$l(\boldsymbol{\theta},\lambda|\mathbf{y}_1,\mathbf{y}_2,...,\mathbf{y}_\mathrm{n}) = \sum_{j=1}^{n}\ln f_{\mathbf{Y}_j|\boldsymbol{\theta}}(\mathbf{y}_j) - \sum_{i=1}^{N}\lambda_i(\boldsymbol{\mu}_i^T\boldsymbol{\mu}_i - r^2). \tag{65}$$

The system of equations to be solved is

$$\begin{cases} \sum_{j=1}^{n}\mathbb{P}(H_j = i|\hat{\boldsymbol{\theta}}){\Sigma_j}^{-1}(\mathbf{Y_j} - \hat{\boldsymbol{\mu}}_i) - 2\lambda_i\hat{\boldsymbol{\mu}}_i = 0 \\ \hat{\boldsymbol{\mu}}_i^T\hat{\boldsymbol{\mu}}_i - r^2 = 0, \end{cases} \tag{66}$$

or, equivalently,

$$\begin{cases} \sum_{j=1}^{n}\mathbb{P}(H_j = i|\hat{\boldsymbol{\theta}})(y_{\mathrm{x},j} - \hat{\mu}_{\mathrm{x},i})/\sigma_{\mathrm{x},ij} - 2\lambda_i\hat{\mu}_{\mathrm{x},i} = 0, \\ \sum_{j=1}^{n}\mathbb{P}(H_j = i|\hat{\boldsymbol{\theta}})(y_{\mathrm{y},j} - \hat{\mu}_{\mathrm{y},i})/\sigma_{\mathrm{y},ij} - 2\lambda_i\hat{\mu}_{\mathrm{y},i} = 0, \\ \sum_{j=1}^{n}\mathbb{P}(H_j = i|\hat{\boldsymbol{\theta}})(y_{\mathrm{z},j} - \hat{\mu}_{\mathrm{z},i})/\sigma_{ij,z} - 2\lambda_i\hat{\mu}_{\mathrm{z},i} = 0, \\ \hat{\mu}_{\mathrm{x},i}^2 + \hat{\mu}_{\mathrm{y},i}^2 + \hat{\mu}_{\mathrm{z},i}^2 - r^2 = 0. \end{cases} \tag{67}$$

$\mu_{\mathrm{x},i}$, $\mu_{\mathrm{y},i}$ and $\mu_{\mathrm{z},i}$ are expressed as a function of λ

$$\begin{cases} \hat{\mu}_{\mathrm{x},i} = \sum_{j=1}^{n}\left(\frac{\mathbb{P}(H_j=i|\hat{\boldsymbol{\theta}})/\sigma_{\mathrm{x},ij}}{\sum_{k=1}^{n}\mathbb{P}(H_k=i|\hat{\boldsymbol{\theta}})/\sigma_{\mathrm{x},ik}+2\lambda_i}\right)y_{\mathrm{x},k}, \\ \hat{\mu}_{\mathrm{y},i} = \sum_{j=1}^{n}\left(\frac{\mathbb{P}(H_j=i|\hat{\boldsymbol{\theta}})/\sigma_{\mathrm{y},ij}}{\sum_{k=1}^{n}\mathbb{P}(H_k=i|\hat{\boldsymbol{\theta}})/\sigma_{\mathrm{y},ik}+2\lambda_i}\right)y_{\mathrm{y},k}, \\ \hat{\mu}_{\mathrm{z},i} = \sum_{j=1}^{n}\left(\frac{\mathbb{P}(H_j=i|\hat{\boldsymbol{\theta}})/\sigma_{\mathrm{z},ij}}{\sum_{k=1}^{n}\mathbb{P}(H_k=i|\hat{\boldsymbol{\theta}})/\sigma_{\mathrm{z},ik}+2\lambda_i}\right)y_{\mathrm{z},k}, \\ \mu_{\mathrm{x},i}^2 + \mu_{\mathrm{y},i}^2 + \mu_{\mathrm{z},i}^2 - r^2 = 0. \end{cases} \tag{68}$$

The first three equations are substituted in the last one to find

$$\frac{(\sum_{j=1}^{n}\mathbb{P}(H_j = i|\hat{\boldsymbol{\theta}})\frac{y_{\mathrm{x},j}}{\sigma_{\mathrm{x},ij}})^2}{(\sum_{j=1}^{n}\mathbb{P}(H_j = i|\hat{\boldsymbol{\theta}})\frac{1}{\sigma_{\mathrm{x},ij}} + 2\lambda_i)^2} + \frac{(\sum_{j=1}^{n}\mathbb{P}(H_j = i|\hat{\boldsymbol{\theta}})\frac{y_{\mathrm{y},j}}{\sigma_{\mathrm{y},ij}})^2}{(\sum_{j=1}^{n}\mathbb{P}(H_j = i|\hat{\boldsymbol{\theta}})\frac{1}{\sigma_{\mathrm{y},ij}} + 2\lambda_i)^2} + \\ \frac{(\sum_{j=1}^{n}\mathbb{P}(H_j = i|\hat{\boldsymbol{\theta}})\frac{y_{\mathrm{z},j}}{\sigma_{\mathrm{z},ij}})^2}{(\sum_{j=1}^{n}\mathbb{P}(H_j = i|\hat{\boldsymbol{\theta}})\frac{1}{\sigma_{\mathrm{z},ij}} + 2\lambda_i)^2} - r^2 = 0. \tag{69}$$

Let us summarize Supplementary Equations 65-69. First the probability to originate from a specific source is assigned to all localizations, based on the assumed emitter locations. Thereafter, estimates of the source locations are updated as a weighted average, taking into account the labelling probability and the uncertainty of the localizations.

For every emitter this process of labelling and updating of the source location is continued until the difference in likelihood for that specific emitter between two subsequent estimation cycles is less than a pre-assigned tolerance, which we take to be 0.1.

Initial positions emitters

The above cycle is very sensitive to the initial estimates of the source locations. A k-means (KM) algorithm is used to provide this initial estimate, see e.g. [14],[15]. According to the KM algorithm, N points are placed randomly in the space of the observations of interest. Next the Euclidean distance for each observation to each of the N points is calculated and an observation is assigned to its closest point. From this, N clusters are obtained and within each cluster one computes the mean of the coordinates of the members of that cluster. The result is a set of N new points and starting from these points the procedure is repeated. The process terminates when the displacement of the new set of points is less than the desired precision. The output of the algorithm is used as the initial estimate for the dye locations. The k-means algorithm makes use of the positions of each localization, but not of the uncertainty (number of photons collected) of the localizations. Since the k-means algorithm starts with a random set of sources, repeating the algorithm on a unique dataset gives different results. Therefore, we run the k-means algorithm 20 (L), 20 (M) and 5 (H) times, respectively, and start with the most likely (highest likelihood) configuration of points as an initial set of emitter positions.

Performance algorithm

It is hard to present an MSE variant for this problem even if the number of emitters would be known. In the latter case the positions of the emitters should be compared one by one. This is a matching problem on a weighted complete bipartite graph. The vertices consist of two equal disjoint sets R and E. Here R represents the real locations of the dyes and E is the set with estimated locations. A weight is assigned to the edge between r and e, $r \in R$ and $e \in E$, equal to the Euclidean distance between these two points. After this, the Hungarian algorithm, see [26, Paragraph 3.5], is used to find a least weighted (perfect) matching for this weighted graph. This perfect matching gives rise to a matching of real and estimated emitter positions. Exploiting this pairing, the distances in the axial and lateral directions between the estimate and the real value are computed. Note that the emitter positions of the real beads are at the core of the problem, they are unknown. However, for the simulated beads these positions are known and hence we compute the distances between estimated and real positions of dyes. In Supplementary Table 11 the average absolute differences between estimates and the real positions in all directions for all bead densities are presented. Furthermore, the resolutions obtained with solely the k-means algorithm is shown. Here it is important to note that we validated the convergence of these quantities based on 1000 simulated beads, see e.g. Supplementary Figures 10-12, from which we conclude that enough simulations were used.

Density	x	KM (x)	y	KM (y)	z	KM(z)
L	17.57	27.43	17.18	27.05	22.08	30.94
M	20.88	23.79	20.47	23.65	22.86	24.97
H	14.72	14.63	14.03	14.63	14.67	15.31

Supplementary Table 11: Average absolute distances in nm for simulated data.

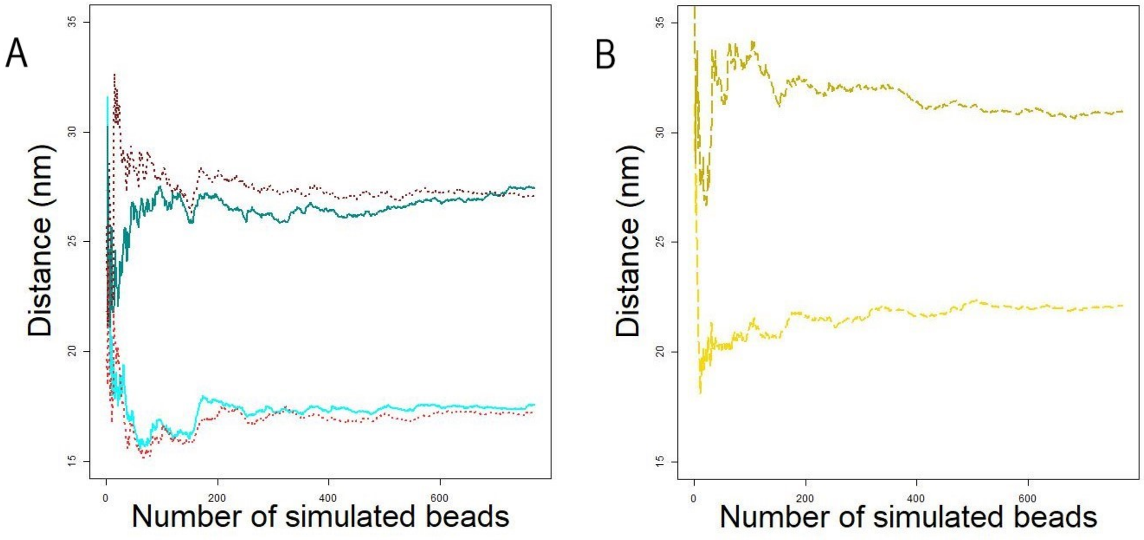

Supplementary Figure 10: Low-density beads convergence plots. a). Lateral resolution obtained with the k-means algorithm in x- (solid pine), respectively y-direction (dashed dark red) and with the proposed algorithm in x- (solid cyan), respectively y-direction (dashed red). b). Axial resolution obtained with the k-means algorithm (dashed gold) and obtained with the proposed algorithm (dashed yellow).

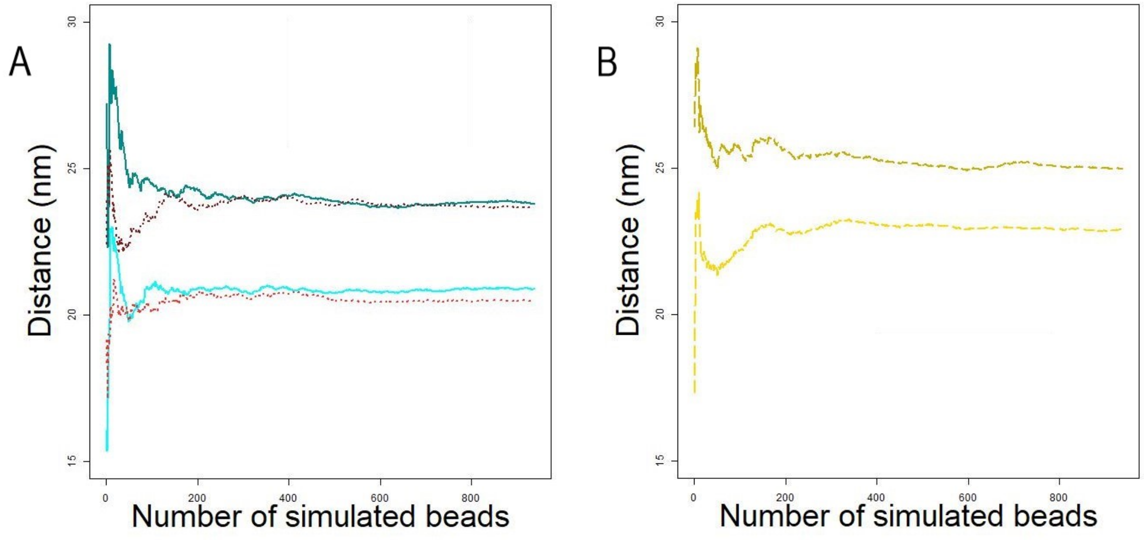

Supplementary Figure 11: Medium-density beads convergence plots. a). Lateral resolution obtained with the k-means algorithm in x- (solid pine), respectively y-direction (dashed dark red) and with the proposed algorithm in x- (solid cyan), respectively y-direction (dashed red). b). Axial resolution obtained with the k-means algorithm (dashed gold) and obtained with the proposed algorithm (dashed yellow).

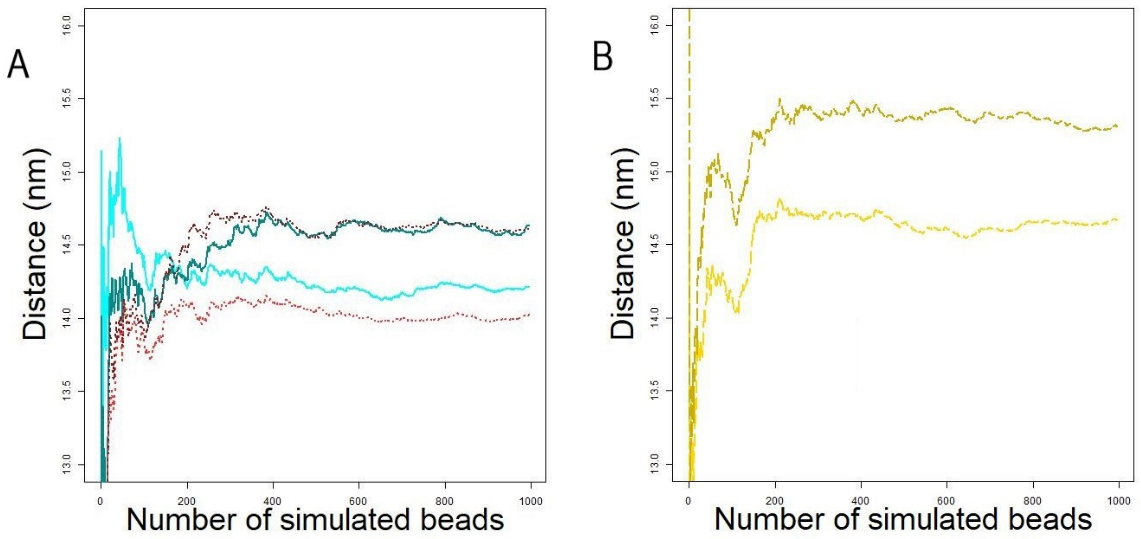

Supplementary Figure 12: High-density beads convergence plots. a). Lateral resolution obtained with the k-means algorithm in x- (solid pine), respectively y-direction (dashed dark red) and with the proposed algorithm in x- (solid cyan), respectively y-direction (dashed red). b). Axial resolution obtained with the k-means algorithm (dashed gold) and obtained with the proposed algorithm (dashed yellow).

Distribution analysis

In subsections and an algorithm that processes dSTORM images to obtain estimates of the number of dyes and of the spatial locations of these dyes has been developed. Next, the assumption that the dyes on a bead behave as a homogeneous negative binomial point process is investigated. To validate this homogeneity assumption the so-called nearest-neighbour distance (NND) is used. The NND equals the arc length of the smallest arc between a dye (estimate) of interest and an arbitrary other dye (estimate) on the same bead. Let $\boldsymbol{e}_i, i = 1, 2, ..., X$ be a dye on a bead with in total X dyes. Notice that all dyes are located on the sphere. The arc-length between two dye locations equals the radius of the bead times the angle between the vectors to the two locations starting at the origin. With the use of the inner product and the inverse cosine, this angle is computed. If $\bullet$ denotes the inner product, then the NND of $\boldsymbol{e}_i$ equals

$$\text{NND}(\boldsymbol{e}_i) = \min_{j \in [1,X] \cap \mathbb{N} \setminus \{i\}} \left\{ \cos^{-1}\left(\frac{\boldsymbol{e}_i \bullet \boldsymbol{e}_j}{|\boldsymbol{e}_i| \cdot |\boldsymbol{e}_j|} \right) \tfrac{1}{\pi} \cdot 165\pi \right\}. \tag{70}$$

Bead-specific clustering quantification

The degree of clustering of a specific bead is a non-trivial concept. dSTORM provides insight into the particle-to-particle variability of functional group distributions. The fluctuation in the degree of clustering that is caused purely by stochasticity is visualized. First the empirical NND distribution based on all NNDs of all real beads of the medium-density is determined. Next, for every NND estimate, a c value is computed that is defined as a percentile with respect to the empirical distribution. Therefore, a high c value indicates that this emitter is closer to a neighbor than an average emitter.

Figure 13 presents two nanoparticles from our dataset, with equal estimated numbers of fluorophores, displaying very different functionalization patterns. The estimated positions are color-coded according to the empirical distribution of NNDs, displaying strongly clustered functional groups on particles in dark red, and weakly clustered particles in yellow and lighter tones.

The main question to be answered is whether the clustering observed in the dataset is likely when assuming 'fully random' functionalization in which functional groups are homogeneously distributed over the beads.

Goodness of fit test

To test whether the (homogeneous) model correctly represents the dye density, the average NND of the estimated dyes on real beads is computed. The test statistic becomes

$$D_1 = \frac{1}{X} \cdot \sum_{i=1}^{X} \text{NND}(\boldsymbol{e}_i) \tag{71}$$

Note that the NND terms depend both on the location of the emitters with respect to each other as well as on the total number of dyes on the bead. This is in agreement with our notion of clustering, that is the total number of functionalized side groups in a certain neighbourhood. The distribution of D_1 based on the homogeneity assumption is approximated by simulating 1000 beads, estimating the number and position of the dyes, and computing the NND of every simulated bead. It is important to mention that the distribution of D_1, and the

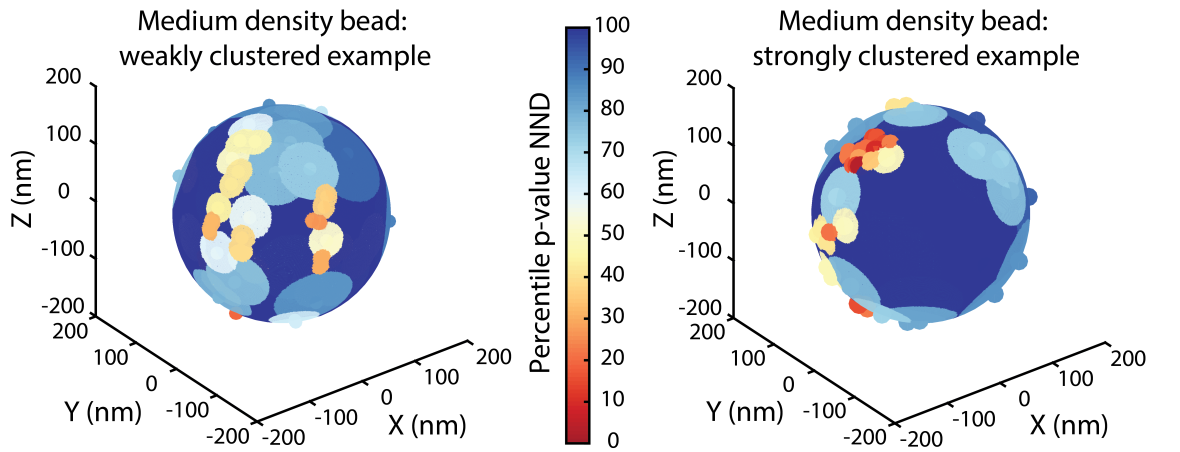

Supplementary Figure 13: Two example (medium-density) nanoparticles analysed using the EM-algorithm, fluorophore location estimates are given as solid points. NND for each fluorophore estimate is graphically depicted as a circle, color-coded according to a p-value (empirical distribution) of that NND in the entire bead population, as indicated in the color bar. For both particles the estimated number of dyes $\hat{n}_{\text{dyes}} = 57$.

distribution of the NND for the simulated emitters, are based on only those simulated beads that satisfy the acquisition constraint presented in Supplementary Table 2 for the different densities. The number of simulated beads used for the empirical distribution n_{sim} is presented in Supplementary Table 12.

Next the hypothesis H_0 that the dyes are uniformly distributed over the bead, is tested for a specific bead. To do so, D_1 is computed for a real bead and the number S of simulated values that are smaller or equal to this D_1 is computed. The outcome of the test D_2 is defined as:

$$D_2 = \mathbb{1}_{\{S \leq 0.025 \cdot n_{\text{sim}}\}} + \mathbb{1}_{\{S \geq 0.975 \cdot n_{\text{sim}}\}} \tag{72}$$

This statistical testing procedure was originally proposed by Dwass in 1957, see [16]. Performing this step for n real beads, see Supplementary Table 1,

$$T = \sum_{i=1}^{n} D_{2,i} \tag{73}$$

is computed. Note that if the model appropriately describes the spatial distribution of the dyes, then T is $\text{Bin}(n, 0.05)$ distributed. To test the hypothesis the p-value $\mathbb{P}(\text{Bin}(n, 0.05) > T)$ is computed. The null hypothesis will be rejected if this probability is less than or equal to 0.05. The critical value, the value of the test statistic and the corresponding p-value for all three densities are shown in Supplementary Table 12.

Bead density	n	n_{sim}	Critical value	Test statistic	p-value
L	274	995	20	30	0.000
M	379	937	26	82	0.000
H	224	769	17	19	0.009

Supplementary Table 12: Homogeneity test results

Based on this test we conclude, for all functionalization-densities taken into account, that the homogeneity assumption is rejected. In addition, qualitative (and practically relevant) arguments in support of the conclusion are presented. In Supplementary Figures 14, 15 and 16 the NND (of all computed NNDs) histograms and CDFs for the estimated locations of the emitters of the real beads and the estimated and real locations of the emitters of the simulated bead are presented.

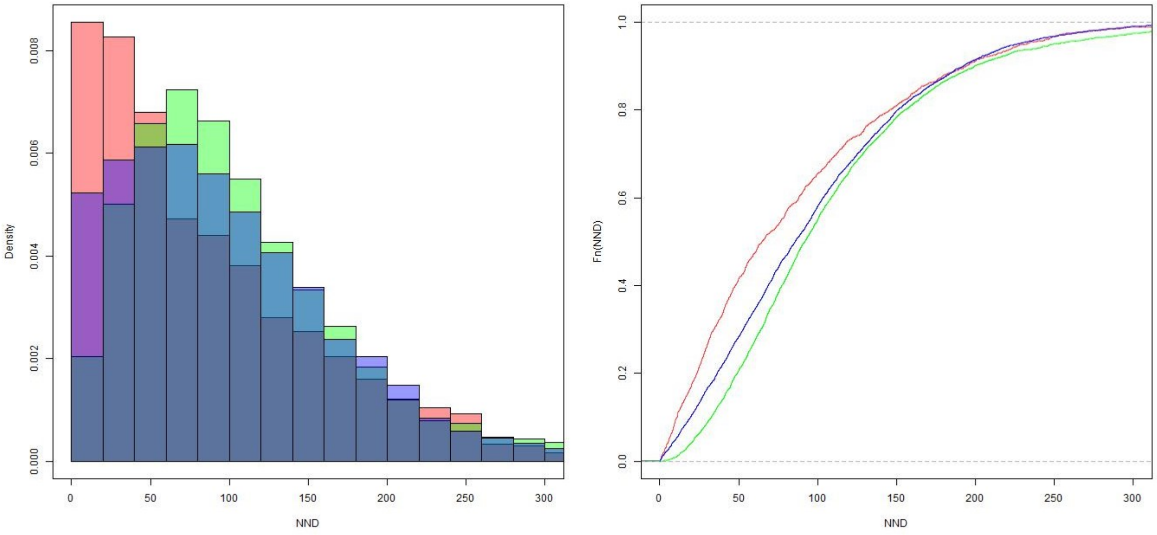

Supplementary Figure 14: NND distribution (histogram left, CDF right) based on estimates for emitter locations of real low-density beads (red) and simulated low-density beads (blue), respectively. In addition, the NND distribution based on the simulated emitter locations is presented (green).

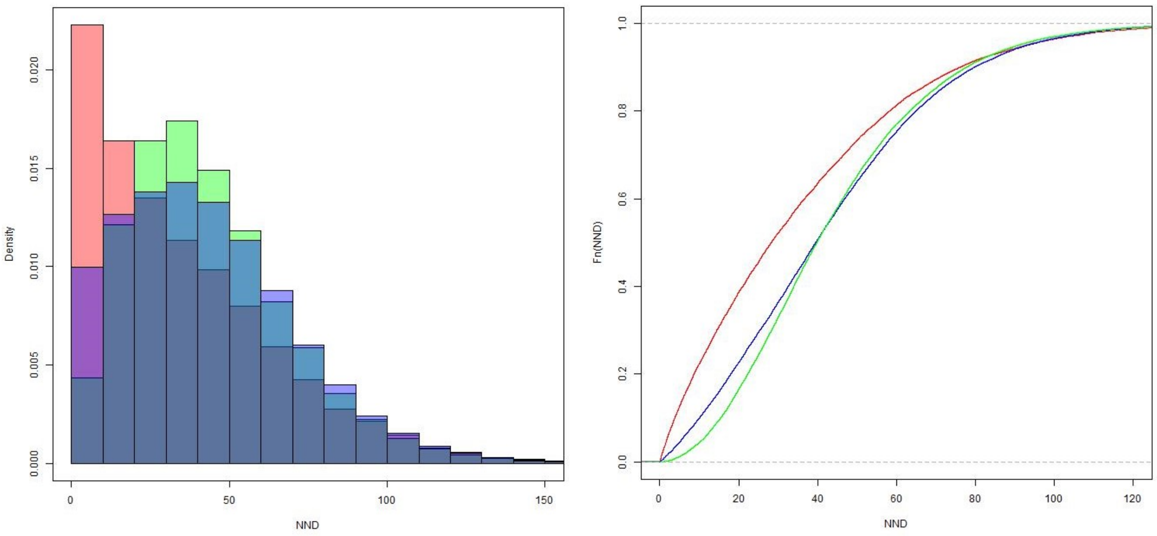

Supplementary Figure 15: NND distribution (histogram left, CDF right) based on estimates for emitter locations of real medium-density beads (red) and simulated medium-density beads (blue), respectively. In addition, the NND distribution based on the simulated emitter locations is presented (green).

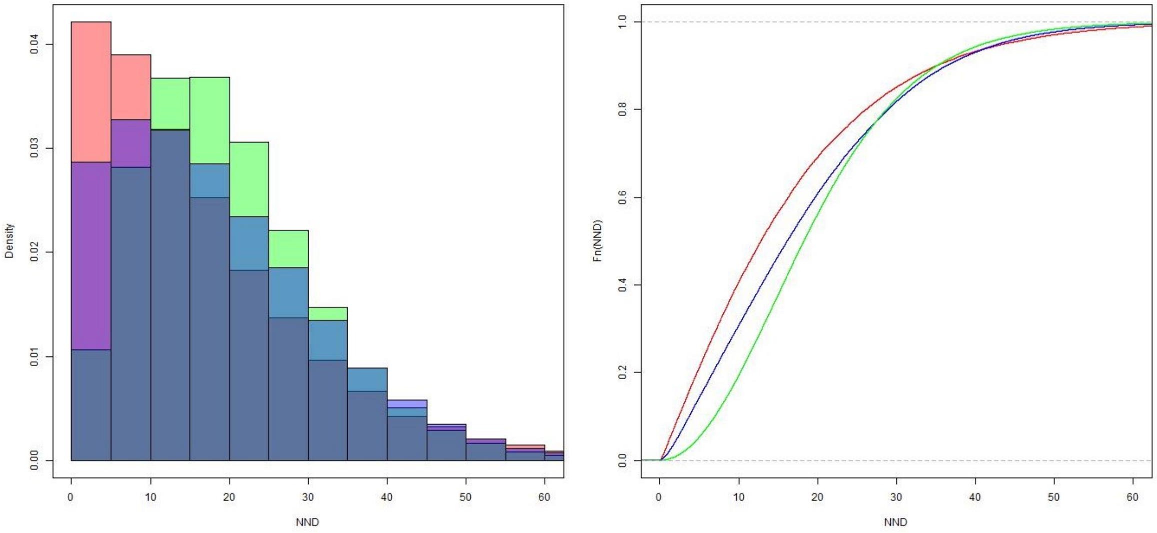

Supplementary Figure 16: NND distribution (histogram left, CDF right) based on estimates for emitter locations of real high-density beads (red) and simulated high-density beads (blue), respectively. In addition, the NND distribution based on the simulated emitter locations is presented (green).

First, for the simulated dyes, we note that the NNDs based on the estimated locations are small in comparison to the NNDs based on the simulated emitter locations. This is explained

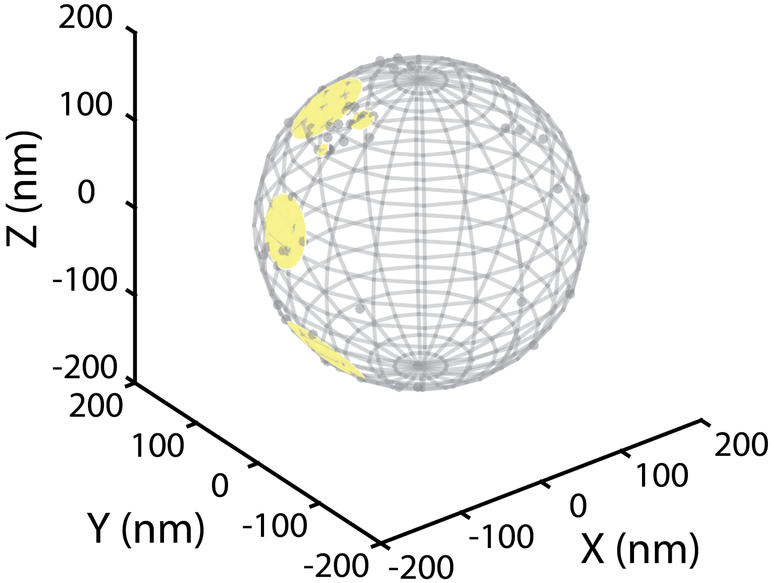

Supplementary Figure 17: Example (5,50)-Cluster midpoints (yellow) based on estimated dyes (grey) of the strongly clustered medium-density bead from Supplementary Figure 13.

by the (reduced) overcounting effect. However, the NND distribution of the dye locations are well described by evaluating the distribution of the estimated positions.

Furthermore, the distribution of the estimated NND from the experimental dataset is compared to the estimated distribution based on the simulations. We conclude that the distribution of the emitters over real beads is not homogeneous. Rather, functional groups on real beads appear to be more clustered than under the homogeneity assumption.

Practical application of the clustering quantification

Based on the estimated number and locations of dyes, insight was gained on various issues connected to clustering. As an example, let us focus on the total number of patches on the surface of the bead that contain at least n emitters with nearest-neighbour distances smaller than k for each emitter in the patch. In this example $n = 5$ and $k = 2 \cdot 50$ nm. In Supplementary Figure 17 an example of an arbitrary (real) medium-density bead that contains 5 such clusters is presented. The empirical distribution of the number of these clusters for the real beads is presented. In addition, the distribution for the simulated beads with emitters distributed homogeneously are evaluated, both based on the real and estimated locations of emitters. Due to the (n, k) choice the low-density beads barely contain clusters. In Supplementary Figures 18 and 19 the histogram and the CDF for both the medium and high-density beads are presented. Hence, we see that inhomogeneity of the functional groups can have large consequences in terms of effect, but the influence of (in)homogeneity depends sensitively on the functionalization density of the beads.

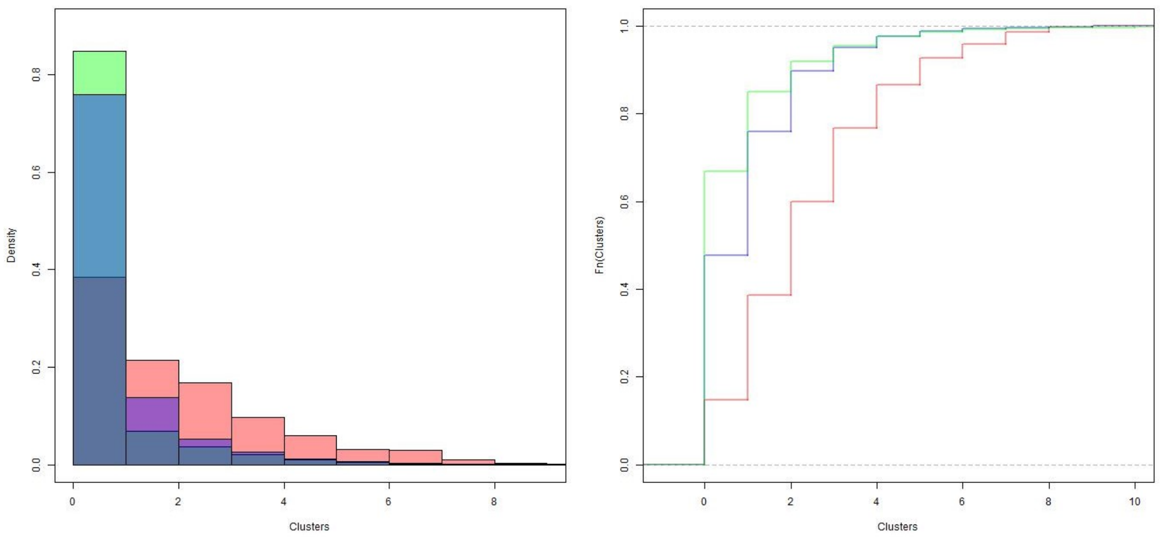

Supplementary Figure 18: (5,50)-cluster distributions (histogram left, CDF right) based on estimates for emitter locations of real medium-density beads (red) and simulated median-density beads (blue), respectively. In addition, the (5,50)-cluster distribution based on the simulated emitter locations is presented (green).

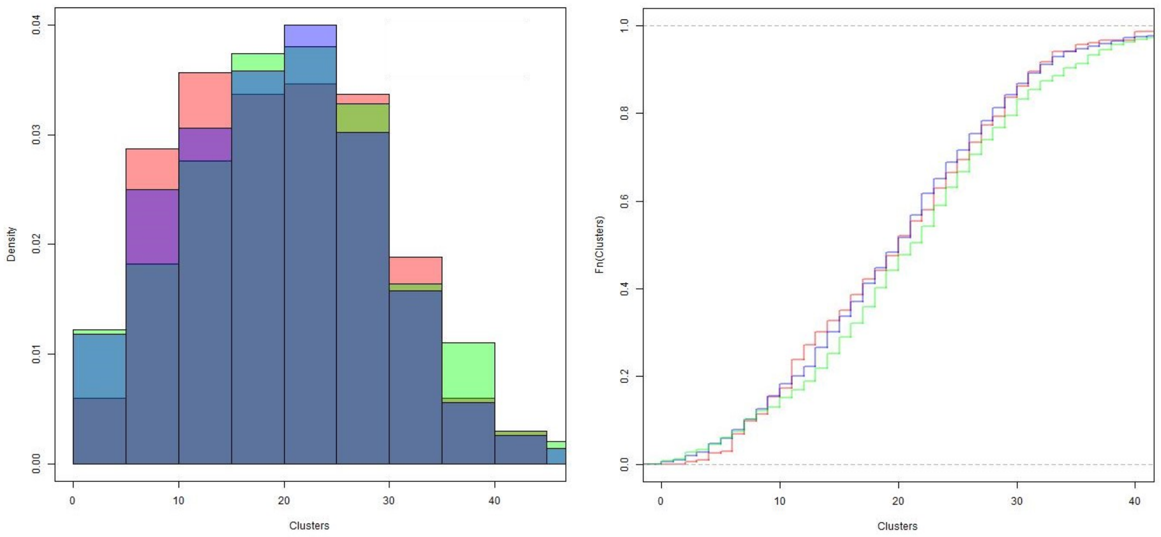

Supplementary Figure 19: (5,50)-cluster distributions (histogram left, CDF right) based on estimates for emitter locations of real high-density beads (red) and simulated high-density beads (blue), respectively. In addition, the (5,50)-cluster distribution based on the simulated emitter locations is presented (green).

Supplementary Note 1

The basic properties of the the probability distributions used in this research are presented in Supplementary Table 13 below.

Distribution	Parameters	$f(x)$	$\mathbb{E}[X]$	$\mathrm{var}(X)$
$\mathcal{N}$	$\boldsymbol{\mu}$, Σ	$\det(2\pi\Sigma)^{-\frac{1}{2}} \exp\left(-\frac{1}{2}(\boldsymbol{x}-\boldsymbol{\mu})'\Sigma^{-1}(\boldsymbol{x}-\boldsymbol{\mu})\right)$	$\boldsymbol{\mu}$	Σ [2]
Exp	λ	$\lambda \exp(-\lambda \cdot x)$	λ^{-1}	λ^{-2}
Geo	p	$(1-p)^{x-1}p$	$\frac{1}{p}$	$\frac{1-p}{p^2}$
Gamma	k, θ	$x^{k-1}\frac{\exp(-x/\omega)}{\omega^k \Gamma(k)}$	$k\theta$	$k\theta^2$
Poi	λ	$\lambda^x \frac{\exp(-\lambda)}{x!}$	λ	λ^2
NB	r, p	$\binom{x+r-1}{x}(1-p)^r p^x$	$\frac{pr}{1-p}$	$\frac{pr}{(1-p)^2}$

Supplementary Table 13: Probability distributions used, see e.g. [20]

In this research we mainly used conditional distributions. Let X be a random variable with probability density function (pdf) or probability mass function (pmf), respectively, $f(x)$ then X restricted to a $a \leq X \leq b$, a subset of the outcome space, has pdf/pmf

$$\tilde{f}(x) = \begin{cases} 0 & \text{if } b \leq x, \\ \frac{f(x)}{F(b)-F(a)} & \text{if } a \leq x \leq b \ , \\ 0 & \text{else} \end{cases} \tag{74}$$

where F represents the cumulative distribution function.

Supplementary Note 2

Poisson point process

$N = (N_A)$, $A \subseteq \mathbb{R}^3$, is a *Poisson Point Process* (PPP) if

1. $$\mathbb{P}(N(A) = 0) = e^{-\int_A \Lambda(x)dx}, \tag{75}$$

   where $\int_A \Lambda(x)dx = \Lambda(A) = \mathbb{E}[N(A)]$, with intensity Λ.

2. $B, C \subseteq A$ and $B \cap C = \emptyset \Rightarrow N(B) \perp N(C) \Leftrightarrow \mathbb{P}(N(B) = 0, N(C) = 0) = \mathbb{P}(N(B) = 0)\mathbb{P}(N(C) = 0)$.

As a result the probability distribution of a PPP equals:

$$\mathbb{P}(N(A) = k) = \frac{\Lambda(A)^k}{k!} \mathrm{e}^{-\Lambda(A)}, \tag{76}$$

see [17].

Let us briefly summarize several crucial properties of the PPP. First of all, the number of points in A, $N(A)$, is a random variable that follows a Poisson distribution with parameter

[2] Σ equals the covariance matrix of the multivariate normal distribution.

$\Lambda(A)$. The points are distributed over A proportionally to the measures of the subsets of A. If the intensity is equal for all subsets of A with the same volume, then the PPP on A is referred to as a homogeneous PPP. For this reason the points that are the realizations of the homogeneous PPP are randomly distributed over A. This means that the distribution of the locations of the points conditioned on the number of points is uniformly distributed over A. PPPs possess important properties, for example, if all the points of a PPP are displaced with an displacement from a known distribution, then the resulting process is again a PPP whose intensity equals the convolution of the intensity of the original PPP with the probability distribution of the displacement, see [18, Example 3.3]. When $N(A)$ is a PPP an important 'thinning' property can be derived. Indeed, let $N^{'}(A) \sim \text{Bin}(N(A), p)$ then $N^{'}(A)$ is again a PPP with intensity $p\Lambda(A)$, see [20, Example 5.1.27].

Markov processes

A Markov process is also referred to as a continuous-time Markov chain. The chain takes values in a finite countable state space for which the time spend in each state is exponentially distributed, see [21, Chapter 6]. The Markov property states that the future behavior of the chain only depends on the current state of the chain. The rate matrix Q is defined as

$$Q_{i,j} = \begin{cases} \lambda_{i,j} & \text{if } i \neq j, \\ -\sum_{j,j\neq i} \lambda_{i,j} & \text{if } i = j, \end{cases} \tag{77}$$

with transition rates $\lambda_{i,j}$. $P(t)$ is the matrix whose ith row represents the probability to be in state $j$at time t if the process starts in state i. The Kolmogorov forward equation state

$$P'(t) = \frac{d}{dt}P(t) = P(t)Q, \tag{78}$$

while the backward equation results in

$$P'(t) = QP(t), \tag{79}$$

see [20, Paragraph 6.9]. If Q is an $n \times n$ matrix with n eigenvectors and $Q = UDU^{-1}$ where U is the matrix of eigenvectors of Q and D the diagonal matrix of eigenvalues. The matrix differential equations are now both solved by

$$P = Ue^{tD}U^{-1}. \tag{80}$$

Note that e^{tD} equals a diagonal matrix of $e^{t\mu_i}$, where μ_i is the ith eigenvalue of Q, for $i \in \{1, ..., n\}$.

Supplementary References

[1] *Lee, S.-H., Shin, J.Y., Lee, A., Bustamante, C.:* Counting single photoactivatable fluorescent molecules by photoactivated localization microscopy (PALM), Proceedings of the National Academy of Sciences of the United States of America, 109 (43) Issue 43, pp. 17436-17441, 2012.

[2] *Nie, S., Chiu, D.T., Zare, R.N.* Probing individual molecules with confocal fluorescence microscopy, Science 266, pp. 1018-1021, 1994.

[3] *Köhn, F., Hofkens, J., Gronheid, R., van der Auweraer, M., de Schryver, F.C.* Parameters influencing the on- and off-times in the fluorescence intensity traces of single cyanine dye molecules, J. Phys. Chem. A 106, pp. 4808-4814, 2002.

[4] *Van de Linde, S., Sauer, M.* How to switch a fluorophore: from undesired blinking to controlled photoswitching, Chem. Soc. Rev. 43, pp. 1076-1087, 2014.

[5] *Dempsey, G.T., Bates, M. Kowtoniuk, W.E., Liu, D.R., Tsien, R.Y., Zhuang, X.* Photoswitching mechanism of cyanine dyes, J. Am. Chem. Soc. 131, pp. 18192-18193, 2009.

[6] *Dempsey, G.T., Vaughan, J.C., Chen, K.H., Bates, M., Zhuang, X.:* valuation of fluorophores for optimal performance in localization-based super-resolution imaging, Nature Methods 8 (12), pp. 1027-1040, 2011.

[7] *Kiessling, L.L., Gestwicki, J.E., Strong, L.E.* Synthetic multivalent ligands as probes of signal transduction, Angew. Chem. Int. Ed. 45, pp. 2348-2368, 2006.

[8] *Thompson, M.A., Lew, M.D., Moerner, W.E.* Extending microscopic resolution with single-molecule imaging and active control, Annual Review of Biophysics, 41 (1), pp. 321-342, 2012

[9] *Holtzer, L., Schmidt, T.*, Single particle tracking and single molecule energy transfer, Chapter 2: The Tracking of Individual Molecules in Cells and Tissues, pp. 25-42, Wiley-VCH, 2010.

[10] *Rust, M.J., Bates, M., Zhuang, X.* Stochastic optical reconstruction microscopy (STORM) provides sub-diffraction-limit image resolution, Nat. Methods 3, pp. 793-795, 2006.

[11] *Huang, B., Wang, W., Bates, M., Zhuang, X.:* Three-dimensional super-resolution imaging by stochastic optical reconstruction microscopy, Science 319 (5864), pp. 810-813, 2008.

[12] *Nikon Instruments Europe B.V.* NIS Elements 4.51 - N-STORM user manual, 2016.

[13] *Wang, W.* Structures and Dynamics in Live Bacteria Revealed by Super-Resolution Fluorescence Microscopy, Harvard University Cambridge, Massachusetts, 2012.

[14] *Duda, R.O., Hart, P.E., Stork, D.G.* Pattern Classification. John Wiley & Sons, 2001.

[15] *Hastie, T., Tibshirani, R., Friedman, J.* The Elements of Statistical Learning Data Mining, Inference, and Prediction. Springer, 2009.

[16] *Dwass, M.* Modified Randomization Tests for Nonparametric Hypotheses, Ann. Math. Statist., Volume 28, Number 1, 181-187, 1957.

[17] *Streit, R.L.* Poisson Point Processes: Imaging, Tracking, and Sensing, Springer-Verlag, New York, 2010.

[18] *Resnick, S.* Extreme Values, Regular Variation, and Point Processes, volume 4 Of Applied Probability. A Series of the Applied Probability Trust, Springer-Verlag, New York, 1987.

[19] *Stallinga, S., Rieger, B.* Accuracy of the Gaussian Point Spread Function model in 2D localization microscopy. Opt. Express 18, pp. 24461-24476, 2010.

[20] *Grimmett, G., Stirzaker, D.* Probability and Random Processes, Oxford University Press, New York, 2001.

[21] *Ross, S.M.* Introduction to Probability Models, Ninth Edition, Academic Press, Inc. Orlando, 2006.

[22] *Mortenson, K.I, Stirling Churchman, L., Spudich, J.A. Flyvbjerg, H.* Optimized localization-analysis for single-molecule tracking and super-resolution microscopy, Nat methods, 7(5), pp.377-381, 2010.

[23] *Sen, P.K., Singer, J.M.* Large sample methods in statistics: an introduction with applications, Chapman & Hall, London, 1993.

[24] *Norris, J.R.*, Markov Chains, Cambridge University Press, Cambridge, 1998.

[25] *van der Vaart, A. W.* Asymptotic Statistics, Cambridge University Press, Cambridge, 1998.

[26] *Schrijver, A.* A Course in Combinatorial Optimization, CWI Amsterdam, 2012.

[27] *Durrett, R.* Probability: Theory and Examples, Version 5a, Cambridge University Press, 2005, `https://services.math.duke.edu/~rtd/PTE/PTEv5a.pdf` (accessed 1 Aug 2017).

[28] *Sachs, L.* Statistische Auswertungsmethoden, 3rd edition., Springer-Verlag, Berlin, 1972.
